# Supplementary material for: Clinical pneumonia in the hospitalised child in Malawi in the post-pneumococcal conjugate vaccine era: a prospective hospital-based observational study
Source: BMJ Open. 2022 Feb 8;12(2):e050188. doi: 10.1136/bmjopen-2021-050188 (PMC8830243; doi:10.1136/bmjopen-2021-050188)
Supplement: Supplementary data [file bmjopen-2021-050188supp002.pdf]

Supplementary Table 2. Full characteristics of children hospitalised with clinical pneumonia, stratified by HIV exposure status

		Group		
Characteristics	All (N=1139)	HEU (N=101)	HUU (N=1038)	p-value
**Demographic and physiological characteristics**				
Age group				
N	1116	99	1017	
0-2 m	126 (11.3)	13 (13.1)	113 (11.1)	
2-6 m	188 (16.9)	23 (23.2)	165 (16.2)	
6-12 m	302 (27.1)	32 (32.3)	270 (26.6)	
12-24 m	318 (28.5)	18 (18.2)	300 (29.5)	
24-60 m	182 (16.3)	13 (13.1)	169 (16.6)	
Low birth weight				0.04*
N	1117	97	1020	
< 2.5 kg (%)	302 (27.0)	35 (36.1)	267 (26.2)	
Weight for height categories				
N	1098	101	998	
<-3 (%)	106 (9.7)	15 (15. 0)	91 (9.1)	
-3- -2 (%)	248 (0.9)	31 (31.0)	217 (21.7)	
>-2 (%)	1102 (98.6)	54 (54.0)	690 (69.1)	
MUAC categories				
N	1139	101	1038	
< 11.5 cm (%)	47 (4.1)	13 (12.9)	34 (3.3)	
Previous visit to health centre				1.00*
N	1139	101	1038	
Yes (%)	979 (86.0)	87 (86.1)	892 (85.9)	
**Household characteristics**				
Household size >4				0.83*
N	1136	101	1035	
Yes (%)	480 (42.3)	44 (43.6)	436 (42.1)	
**Clinical features**				
Crackles				0.75*
N	1139	101	1038	
Yes (%)	623 (54.7)	57 (56.4)	566 (54.5)	
Wheezing				0.41*
N	1137	101	1036	
Yes (%)	192 (16.9)	20 (19.8)	172 (16.6)	
Bronchial breathing				0.12*
N	1139	101	1038	
Yes (%)	70 (6.2)	10 (9.9)	60 (5.8)	
Reduced air entry				0.19*
N	1139	101	1038	

Yes	9 (0.8)	2 (2.0)	7 (0.7)	
Age-adjusted tachycardia				0.11*
N	505	46	459	
Age 0-12m and >160 beats/min	266 (52.7)	31 (67.4)	235 (51.2)	
Age 12-36m and >150 beats/min	204 (40.4)	14 (30.4)	190 (41.4)	
Age 36-60m and >140 beats/min	35 (6.9)	1 (2.2)	34 (7.4)	
Hepatomegaly				0.61*
N	1135	99	1036	
Yes (%)	11 (1.0)	0 (0.0)	11 (1.1)	
Oxygen saturation				0.28†
N	1128	100	1028	
Median (IQR)	95.0 (89.0, 98.0)	95.0 (86.0, 98.0)	95.0 (89.8, 98.0)	
**Clinical management**				
Received IVF				1.00*
N	1120	100	1020	
Yes (%)	14 (1.3)	1 (1.0)	13 (1.3)	

HEU, HIV-exposed, uninfected; HUU, HIV-unexposed, uninfected; IQR, interquartile range;

IVF, intravenous fluids; MUAC, mid-upper arm circumference

** Fisher's exact test*

† Wilcoxon rank sum test
