# Supplementary material for: Clinical pneumonia in the hospitalised child in Malawi in the post-pneumococcal conjugate vaccine era: a prospective hospital-based observational study
Source: BMJ Open. 2022 Feb 8;12(2):e050188. doi: 10.1136/bmjopen-2021-050188 (PMC8830243; doi:10.1136/bmjopen-2021-050188)
Supplement: Author's manuscript [file bmjopen-2021-050188.draft_revisions.pdf]

BMJ Open is committed to open peer review. As part of this commitment we make the peer review history of every article we publish publicly available.

When an article is published we post the peer reviewers' comments and the authors' responses online. We also post the versions of the paper that were used during peer review. These are the versions that the peer review comments apply to.

The versions of the paper that follow are the versions that were submitted during the peer review process. They are not the versions of record or the final published versions. They should not be cited or distributed as the published version of this manuscript.

BMJ Open is an open access journal and the full, final, typeset and author-corrected version of record of the manuscript is available on our site with no access controls, subscription charges or pay-per-view fees (http://bmjopen.bmj.com).

If you have any questions on BMJ Open's open peer review process please email info.bmjopen@bmj.com

BMJ Open

Clinical pneumonia in the hospitalised child in Malawi in the post-PCV era

Journal:	*BMJ Open*
Manuscript ID	bmjopen-2021-050188
Article Type:	Original research
Date Submitted by the Author:	12-Feb-2021

Complete List of Authors:	Iroh Tam, Pui-Ying ; Malawi-Liverpool-Wellcome Trust Clinical Research Programme, Department of Paediatrics and Child Health; Liverpool School of Tropical Medicine, Department of Paediatrics and Child Health<br>Chirombo, James; Malawi-Liverpool-Wellcome Trust Clinical Research Programme<br>Henrion, Marc; Malawi-Liverpool-Wellcome Trust Clinical Research Programme; Liverpool School of Tropical Medicine,<br>Newberry, Laura; Malawi-Liverpool-Wellcome Trust Clinical Research Programme, Department of Paediatrics and Child Health<br>Mambule, Ivan; Malawi-Liverpool-Wellcome Trust Clinical Research Programme<br>Everett, Dean; Malawi-Liverpool-Wellcome Trust Clinical Research Programme, Department of Paediatrics and Child Health<br>Mwansambo, Charles; Government of Malawi Ministry of Health<br>Cunliffe, Nigel; University of Liverpool, Clinical Infection, Microbiology and Immunology<br>French, Neil; University of Liverpool Faculty of Health and Life Sciences, Centre for Global Vaccine Research, Institute of Infection and Global Health<br>Heyderman, Robert; University College London, Division of Infection and Immunity<br>Bar-Zeev, Naor ; Johns Hopkins University Bloomberg School of Public Health, Global Disease Epidemiology and Contro

Keywords:	Epidemiology < INFECTIOUS DISEASES, Paediatric infectious disease & immunisation < PAEDIATRICS, Respiratory infections < THORACIC MEDICINE

SCHOLARONE™
Manuscripts

I, the Submitting Author has the right to grant and does grant on behalf of all authors of the Work (as defined in the below author licence), an exclusive licence and/or a non-exclusive licence for contributions from authors who are: i) UK Crown employees; ii) where BMJ has agreed a CC-BY licence shall apply, and/or iii) in accordance with the terms applicable for US Federal Government officers or employees acting as part of their official duties; on a worldwide, perpetual, irrevocable, royalty-free basis to BMJ Publishing Group Ltd ("BMJ") its licensees and where the relevant Journal is co-owned by BMJ to the co-owners of the Journal, to publish the Work in this journal and any other BMJ products and to exploit all rights, as set out in our licence.

The Submitting Author accepts and understands that any supply made under these terms is made by BMJ to the Submitting Author unless you are acting as an employee on behalf of your employer or a postgraduate student of an affiliated institution which is paying any applicable article publishing charge ("APC") for Open Access articles. Where the Submitting Author wishes to make the Work available on an Open Access basis (and intends to pay the relevant APC), the terms of reuse of such Open Access shall be governed by a Creative Commons licence – details of these licences and which Creative Commons licence will apply to this Work are set out in our licence referred to above.

Other than as permitted in any relevant BMJ Author's Self Archiving Policies, I confirm this Work has not been accepted for publication elsewhere, is not being considered for publication elsewhere and does not duplicate material already published. I confirm all authors consent to publication of this Work and authorise the granting of this licence.

For peer review only

Clinical pneumonia in the hospitalised child in Malawi in the post-PCV era

Pui-Ying Iroh Tam,[1,2,3,a*] James Chirombo,[4*] Marc Y. R. Henrion,[3,4] Laura Newberry,[1,2,b] Ivan Mambule,[1,5,c] Dean Everett,[1,5] Charles Mwansambo,[6] Nigel A. Cunliffe,[5] Neil French,[5] Robert S. Heyderman,[7] and Naor Bar-Zeev[1,2,5,d] for the VacSurv Consortium

[1]Paediatrics and Child Health Research Group, Malawi-Liverpool-Wellcome Trust Clinical Research Programme, Blantyre, Malawi

[2]Department of Paediatrics and Child Health, University of Malawi College of Medicine, Blantyre, Malawi

[3]Department of Clinical Sciences, Liverpool School of Tropical Medicine, Liverpool, UK

[4]Statistical Support Unit, Malawi-Liverpool-Wellcome Trust Clinical Research Programme, Blantyre, Malawi

[5]Institute of Infection, Veterinary and Ecological Science, University of Liverpool, Liverpool, UK

[6]Malawi Ministry of Health, Lilongwe, Malawi

[7]Research Department of Infection, Division of Infection & Immunity, University College London, London, UK

*These authors contributed equally to the manuscript.

[a]Corresponding author: irohtam@mlw.mw

[b]Current address: Kaiser Permanente, Parker, CO, USA

[c]Current address: Medical Research Council, Kampala, Uganda

For peer review only

[d]Current address: Johns Hopkins School of Public Health, Baltimore, MD, USA

Short title: Clinical pneumonia in the Malawian child

Abbreviations

CI, confidence interval

CPAP, continuous positive airway pressure

HEU, HIV-exposed, uninfected

HUU, HIV-unexposed, uninfected

IMCI, integrated management of childhood illness

IQR, interquartile range

mRISC, modified respiratory index of severity in children

MUAC, mid-upper arm circumference

PCV13, 13-valent pneumococcal conjugate vaccine

ResViNet, Respiratory Syncytial Virus Network

RISC, respiratory index of severity in children

For peer review only

For peer review only - http://bmjopen.bmj.com/site/about/guidelines.xhtml

Abstract

Objective

Assess characteristics of clinical pneumonia after introduction of pneumococcal conjugate vaccine (PCV), by HIV exposure status.

Methods and findings

We evaluated 1,139 children ≤5 years old hospitalised with clinical pneumonia: 101 HIV-exposed uninfected (HEU) and 1038 HIV-unexposed, uninfected (HUU). Median age was 11 months, 59% were male, median MUAC was 14 cm (IQR 13-15) and mean weight-for-height z score was -0.7 (±2.5). The highest Respiratory Index of Severity in Children (RISC) scores were allocated to 10.4% of the overall cohort, respectively. Only 45.7% had fever, and 37.2% had at least one danger sign at presentation. The most common clinical features were crackles (54.7%), nasal flaring (53.5%), and lower chest wall indrawing (53.2%). Compared to HUU, HEU children were significantly younger (9 months v. 11 months), with lower mean birth weight (2.8 kg v. 3.0 kg) and MUAC (13.6 cm v. 14.0 cm), had higher prevalence of vomiting (32.7% v. 22.0%), tachypnoea (68.4% v. 49.8%), and highest RISC scores (20.0% v. 9.4%). However, clinical outcomes were similar for both groups.

Conclusions

In this post-PCV setting where prevalence of HIV and malnutrition is high, children hospitalised fulfilling the WHO IMCI criteria for clinical pneumonia present with heterogeneous features. These vary by HIV exposure status but this does not influence either the frequency of danger signs or mortality. The poor performance of available severity scores in this population and the

For peer review only - http://bmjopen.bmj.com/site/about/guidelines.xhtml

absence of more specific diagnostics hinder appropriate antimicrobial stewardship and the rational application of other interventions.

244 words (max 250)

Keywords: children, HIV-exposed, pneumonia, predictive value, respiratory tract, risk score

For peer review only

Strengths and limitations of the study

- We evaluated over 1,100 children hospitalized with pneumonia in a low-income country setting after introduction of PCV.
- Data were sufficient to calculate RISC scores, which performed poorly in this population.
- Overall mortality was low in our cohort and therefore we were unable to evaluate predictors of severe pneumonia.
- Our findings in this setting where prevalence of HIV and malnutrition is high, were that children hospitalised fulfilling the WHO IMCI criteria for clinical pneumonia in the post-PCV era present with heterogeneous features, which hinders appropriate antimicrobial stewardship and the rational application of other interventions.

For peer review only - http://bmjopen.bmj.com/site/about/guidelines.xhtml

Introduction

Pneumonia is the leading cause of morbidity and mortality in children globally. This disease is of particular importance in low-income countries such as Malawi, where HIV prevalence among pregnant women is 10.8% [1], and where a high burden of malaria and malnutrition (stunting prevalence of 39% [2]) contributes to an under-5 mortality rate of 55 per 1,000 live births [3].

WHO Integrated Management of Childhood Illness (IMCI) guidelines for community and referral level management of children with pneumonia rely upon clinical recognition of features of severe disease. WHO lists general danger signs, such as inability to drink, persistent vomiting, convulsions, lethargy or unconsciousness, or stridor in a calm child, which are clinical features used to identify children who should be treated in an inpatient setting and to receive injectable antibiotics [4]. However, clinical features are non-specific and can reflect a variety of other infectious (e.g. bronchiolitis, malaria, sepsis, tuberculosis, pneumocystis), and non-infectious (e.g. asthma, lymphocytic interstitial pneumonitis, pulmonary Kaposi sarcoma) conditions. These have implications for antimicrobial stewardship in the post-pneumococcal conjugate vaccine (PCV) era, particularly in low-resource settings where antimicrobial choice and supply is limited and antimicrobial resistance rates are rising [5].

Few risk scoring systems to predict severe pneumonia exist that are adaptable to low-income countries with high HIV prevalence – Respiratory Index of Severity in Children-Malawi (RISC-Malawi) is the only score from children from a low-income country, and did not consider HIV status. Furthermore, although the alternative, Respiratory Index of Severity in Children (RISC) score does include a scoring system based on HIV infection, no studies have specifically

For peer review only

For peer review only - http://bmjopen.bmj.com/site/about/guidelines.xhtml

evaluated whether HIV exposure status affects the presence and predictive value of clinical features for severe disease and case-fatality. As HIV-exposed, uninfected (HEU) children constitute a growing population and, compared to HIV-unexposed, uninfected (HUU) remain vulnerable to more severe pneumonia, more often fail empiric pneumonia treatment, and have higher rates of hospitalisation and death than HIV-unexposed, uninfected (HUU) children [6-9], this population is of growing clinical relevance. Our objectives were to describe the clinical features of children hospitalised with pneumonia, calculate risk scores for our population, and to determine whether differences in clinical characteristics and calculated risk scores exist by HIV exposure status.

Methods

This cohort was nested within a prospective hospital-based study of 13-valent PCV effectiveness conducted at Queen Elizabeth Central Hospital between 2 April 2013 and 3 August 2016. Children born on or after 1 October 2012 and therefore eligible for PCV and admitted with a diagnosis of clinical pneumonia were enrolled. Children were excluded if they had known oncological or congenital heart disease, were admitted ≥48 hours before recruitment, were re-admitted to hospital within 14 days of a previous hospitalisation, or presented moribund with impending death. Study staff performed active case finding Monday-Friday during daytime in the emergency department and inpatient wards, and followed up study participants 6 weeks following hospital discharge with a home visit or phone call to confirm vital status.

With informed written parental consent, all enrolled children had baseline characteristics measured which included clinical, laboratory and sociodemographic variables. Oxygen

For peer review only

saturation was measured with an appropriately sized sensor and a Nellcor pulse oximeter (Welch Allyn Spot Vital Signs Devices, Skaneateles Falls, NY, USA) in room air. Children with oxygen saturations <90% in room air were given supplemental oxygen via nasal cannula using an oxygen concentrator. At clinician decision and if a free circuit was available, children with severe respiratory distress, signs of exhaustion or worsening hypoxemia received bubble continuous positive airway pressure (Pumani, 3rd Stone Design, San Rafael, CA, USA). Blood was routinely collected for packed cell volume and malaria parasite thick film. Mothers and infants were tested for HIV according to the Malawi National HIV Guidelines [10]. A blood culture was obtained only when the admitting clinician suspected sepsis. Children with suspected meningitis, malaria or anaemia, were managed according to respective WHO and hospital treatment guidelines [11].

This study was approved by the University of Malawi College of Medicine Research Ethics Committee (P.05/13/1386) and by the University of Liverpool (RETH490). Patients or the public were not involved in the design, or conduct, or reporting, or dissemination plans of our research.

Case definition

Pneumonia was diagnosed clinically per WHO IMCI criteria.

Statistical analysis

We assessed the demographic and clinical characteristics of clinical pneumonia patients and compared HEU vs. HUU children using Fisher's exact test, two-sample t-test or Wilcoxon rank-sum tests respectively for categorical, continuous normal and continuous skewed variables.

For peer review only - http://bmjopen.bmj.com/site/about/guidelines.xhtml

We computed RISC scores for severe pneumonia in our cohort [12-14], using the variables listed in **Error! Reference source not found.** 1. We had insufficient variables to compute modified Respiratory Index of Severity in Children (mRISC) and RISC-Malawi scores [13].

Results

A total of 1660 children were recruited into the study, among whose files were incomplete for 521. We included 1,139 children in the analysis, of whom 101 were HEU and 1,038 HUU. The median age was 11 months, 59% were male, and the median mid-upper arm circumference (MUAC) was 14 cm (IQR 13-15) and mean weight-for-height z score -0.7 (±2.5; Table 2).

In our cohort, no single clinical feature was present in more than 55% of the population: the most common clinical features were crackles (54.7%), nasal flaring (53.5%), and lower chest wall indrawing (53.2%). Less than half had fever (45.7%), and at least one IMCI danger sign (37.2%). The median oxygen saturation at presentation was 95% (IQR 89,98), with 25.4% having oxygen saturations below 90%. Among this cohort, 33.9% received oxygen and 2.1% were placed on CPAP. The highest RISC scores (3+) were allocated to 10.4% of the overall cohort, respectively. Five children died (0.4%).

Ten percent of children in our cohort were HEU, and notable differences between HEU and HUU were observed in several areas. In the HEU group, children were younger (median age 9 months v. 11 months, p=0.02), with lower birth weight (median 2.8 kg (IQR 1.8-3.2) v. 3.0 kg (IQR 2.4-3.3), p=0.03), lower weight-for-height z score (mean -1.2±2.5 vs. -0.6±2.5, p<0.01) and

For peer review only - http://bmjopen.bmj.com/site/about/guidelines.xhtml

lower MUAC (median 13.6 cm (IQR 12.6-14.6) v. 14 cm (IQR 13-15), p<0.01). Compared to the HUU group, HEU children had significantly higher prevalence of vomiting (32.7% v. 22.0%, p=0.02) and age-defined tachypnoea in infancy (68.4% v. 49.8%, p=0.02). More HEU children presented with at least one IMCI danger sign (46.5% v. 36.3%, p=0.05). We did not observe significant differences in respiratory symptoms and in proportions of children who died between groups, although HEU children received oxygen at a significantly higher rate than HUU children (44.0% v. 32.9%, p=0.04). Clinical outcomes between the HEU and HUU groups were also similar. However, RISC scores were significantly different between HIV exposure status, with 20.0% of HEU with the highest scores, compared to 9.4% of HUU (Fig 1). The risk score had a nonparametric distribution and many subjects scored 0.

Discussion

In our prospective cohort of children presenting with WHO IMCI criteria for clinical pneumonia in the post-PCV era, the most common clinical features were crackles, nasal flaring, and lower chest wall indrawing. Despite the clinical diagnosis of pneumonia, features were heterogeneous. Thirty-four percent had an oxygen requirement and 2.1% needed CPAP support, though with best available treatment, overall mortality was low – and possibly a reflection of improvements in child health overall as a result of antiretroviral and antimalarial therapies, vaccination, and community management of nutrition. Ten percent of children hospitalised with pneumonia were HEU and this group was significantly younger, with lower birth weight and anthropometric parameters. HEU children had a significantly higher prevalence of vomiting, age-defined tachypnoea in infancy, and higher RISC scores, compared to HUU children.

For peer review only - http://bmjopen.bmj.com/site/about/guidelines.xhtml

Though differences in age and anthropometry were noted, these were subtle and may not be useful for a clinician, who in low resource settings make decisions primarily on clinical suspicion. WHO IMCI clinical criteria for pneumonia are non-specific, and based on presence of cough, fast and difficult breathing. The non-specific nature of the diagnosis, which covers both infectious and non-infectious causes, is deliberately intended to increase sensitivity; however, as reflected in this study, this may have resulted in the term being used with a variety of features. Much research has been expended on developing pneumonia risk scores to identify children at risk of severe disease. However, the limitations of existing pneumonia risk scores for children in low- and middle-income country settings [15], including Pneumonia Etiology Research in Child Health (PERCH) [16], Respiratory Syncytial Virus Network (ReSViNet) [17], RISC [14], mRISC [12], and RISC-Malawi [13], is that the predictive capacity is generally modest and, in the case of PERCH, was found to be not as discriminatory as the WHO danger signs. The risk scores we calculated in our cohort were unevenly distributed and yielded surprisingly divergent results, indicating that they are limited to their study populations and are not generalisable.

In our cohort, the lack of predominating features of clinical pneumonia points to another possibility that, in post-PCV settings where HIV and chronic malnutrition rates are high (10% and 39%, respectively), children do not present with typical signs and symptoms. The difference in distribution of risk scores within our cohort highlights the fact that risk scores are population- and cohort-specific. In our cohort a substantial proportion of children met criteria for severe acute malnutrition (9.7% had weight-for-height z scores ≤3; and 4.1% had MUAC <11.5 cm), indicating how widespread chronic malnutrition is in Malawi. This pervasive underlying comorbidity leads to subsequent issues with identification and management of disease; and may

For peer review only

For peer review only - http://bmjopen.bmj.com/site/about/guidelines.xhtml

in part explain overuse of antimicrobials in this setting, one factor contributing to an increase in antimicrobial resistance.

Antimicrobial stewardship and infection control issues in low-resource settings have been particularly problematic among young children, with significant increases in antimicrobial resistance noted among young infants with bloodstream infections over a twenty-year period [5], and concerns over resistance rates in childhood pneumonia in sub-Saharan Africa [18]. Challenges with diagnostic capacity and the limitations of clinical diagnosis hinder appropriate antimicrobial stewardship [19]. Risk scores that are adequately discriminatory therefore present an opportunity for targeted interventions, including early aggressive support, as well as judicious allocation of resources.

This study has several limitations. Only a very small proportion of HIV-exposed infants had HIV status tested by PCR, as recommended by national guidelines. This was due to shortage of HIV counselors, lack of testing reagents and reluctance of parents to obtain additional testing on their children. We did not recruit moribund children who died very soon after arrival, and therefore we may have underestimated our fatality rate. A substantial proportion of recruited participants had incomplete files and were therefore not included in the analysis. Due to small numbers, we were unable to calculate predictive values for severe outcomes. However, this was a detailed prospective study conducted in a low resource setting in the post-PCV era where robust diagnostic testing was available.

For peer review only - http://bmjopen.bmj.com/site/about/guidelines.xhtml

In conclusion, in this setting where prevalence of HIV and malnutrition is high, children hospitalised fulfilling the WHO IMCI criteria for clinical pneumonia in the post-PCV era present with heterogeneous features. These vary by HIV exposure status but this does not influence either the frequency of danger signs or mortality. The poor performance of available severity scores in this population and the absence of more specific diagnostics hinder appropriate antimicrobial stewardship and the rational application of other interventions. Further work in developing antimicrobial stewardship scores in resource-limited settings, in a period defined by rising rates of antimicrobial resistance, may be warranted.

1,845 words

For peer review only

Acknowledgements

The authors wish to thank the collaborating members of the VacSurv Consortium (James Beard, Amelia C Crampin, Carina King, Sonia Lewycka, Hazzie Mvula, Tambosi Phiri, Miren Iturriza-Gomara, Osamu Nakagomi, Jennifer Verani, Cynthia Whitney). This work was supported by a Wellcome Trust Programme Grant (Grant number 091909/Z/10/Z) and the MLW Programme Core Award (Grant 206454) from the Wellcome Trust. The funders had no role in study design, data collection and analysis, decision to publish, or preparation of the manuscript. The findings and conclusions in this report are those of the authors and do not necessarily represent the official position of the Centers for Disease Control and Prevention, Atlanta USA.

Financial Disclosure

PI has received investigator-initiated research grant support from Bill & Melinda Gates Foundation outside the scope of this work; NAC has received research grant support and honoraria for participation in rotavirus vaccine advisory board meetings from GlaxoSmithKline Biologicals and from Takeda Pharmaceuticals outside the scope of this work; NF has received investigator-initiated research grant support from GlaxoSmithKline Biologicals and from Takeda Pharmaceuticals outside the scope of this work. NBZ has received investigator-initiated research grant support from GlaxoSmithKline Biologicals, Takeda Pharmaceuticals, Merck-Sharpe-Dohme and the Serum Institute of India, all outside the scope of this work. All other authors declare that they have no financial disclosures.

Competing interests

All authors have no competing interests.

For peer review only

For peer review only - http://bmjopen.bmj.com/site/about/guidelines.xhtml

Author contributorship statement

Conceived and designed the research question: PI, NBZ; Conducted the statistical analysis: JC, MH; Managed the study: LN, IM; Developed and awarded funding for the study: DE, NAC, CM, NF, RH; Wrote the first draft of the manuscript: PI; Provided critical feedback on the manuscript: JC, MH, NAC, NF, RH, NBZ; Reviewed and approved the final draft: all authors.

Data availability

Data is available upon reasonable request to the corresponding author.

For peer review only

References

1. UNAIDS: **Malawi factsheet**. 2019.
2. World Bank: **Prevalence of stunting - Malawi**. 2018.
3. WHO: **Malawi Maternal and Child Health Data**. Geneva, Switzerland: WHO; 2015.
4. WHO: **Revised WHO classification and treatment of childhood pneumonia at health facilities**. Geneva, Switzerland: WHO; 2014.
5. Iroh Tam PY, Musicha P, Kawaza K, Cornick J, Denis B, Freyne B, Everett D, Dube Q, French N, Feasey N *et al*: **Emerging Resistance to Empiric Antimicrobial Regimens for Pediatric Bloodstream Infections in Malawi (1998-2017)**. *Clin Infect Dis* 2019, **69**(1):61-68.
6. Landes M, van Lettow M, Chan AK, Mayuni I, Schouten EJ, Bedell RA: **Mortality and health outcomes of HIV-exposed and unexposed children in a PMTCT cohort in Malawi**. *PLoS One* 2012, **7**(10):e47337.
7. Slogrove A, Reikie B, Naidoo S, De Beer C, Ho K, Cotton M, Bettinger J, Speert D, Esser M, Kollmann T: **HIV-exposed uninfected infants are at increased risk for severe infections in the first year of life**. *J Trop Pediatr* 2012, **58**(6):505-508.
8. Slogrove AL, Goetghebuer T, Cotton MF, Singer J, Bettinger JA: **Pattern of Infectious Morbidity in HIV-Exposed Uninfected Infants and Children**. *Front Immunol* 2016, **7**:164.
9. Zar HJ, Barnett W, Stadler A, Gardner-Lubbe S, Myer L, Nicol MP: **Aetiology of childhood pneumonia in a well vaccinated South African birth cohort: a nested

For peer review only - http://bmjopen.bmj.com/site/about/guidelines.xhtml

case-control study of the Drakenstein Child Health Study. *Lancet Respir Med* 2016, **4**(6):463-472.

10. Malawi Ministry of Health: **Clinical Management of HIV in Adults and Children**. Malawi; 2011.

11. WHO: **Handbook IMCI. Integrated management of childhood illness**. In. Geneva, Switzerland: World Health Organization; 2000.

12. Emukule GO, McMorrow M, Ulloa C, Khagayi S, Njuguna HN, Burton D, Montgomery JM, Muthoka P, Katz MA, Breiman RF *et al*: **Predicting mortality among hospitalized children with respiratory illness in Western Kenya, 2009-2012**. *PLoS One* 2014, **9**(3):e92968.

13. Hooli S, Colbourn T, Lufesi N, Costello A, Nambiar B, Thammasitboon S, Makwenda C, Mwansambo C, McCollum ED, King C: **Predicting Hospitalised Paediatric Pneumonia Mortality Risk: An External Validation of RISC and mRISC, and Local Tool Development (RISC-Malawi) from Malawi**. *PLoS One* 2016, **11**(12):e0168126.

14. Reed C, Madhi SA, Klugman KP, Kuwanda L, Ortiz JR, Finelli L, Fry AM: **Development of the Respiratory Index of Severity in Children (RISC) score among young children with respiratory infections in South Africa**. *PLoS One* 2012, **7**(1):e27793.

15. Deardorff KV, McCollum ED, Ginsburg AS: **Pneumonia Risk Stratification Scores for Children in Low-Resource Settings: A Systematic Literature Review**. *Pediatr Infect Dis J* 2018, **37**(8):743-748.

16. Gallagher KE, Knoll MD, Prosperi C, Baggett HC, Brooks WA, Feikin DR, Hammitt LL, Howie SRC, Kotloff KL, Levine OS *et al*: **The Predictive Performance of a**

For peer review only

For peer review only - http://bmjopen.bmj.com/site/about/guidelines.xhtml

Pneumonia Severity Score in Human Immunodeficiency Virus-negative Children Presenting to Hospital in 7 Low- and Middle-income Countries. *Clin Infect Dis* 2020, **70**(6):1050-1057.

17. Justicia-Grande AJ, Pardo-Seco J, Cebey-Lopez M, Vilanova-Trillo L, Gomez-Carballa A, Rivero-Calle I, Puente-Puig M, Curros-Novo C, Gomez-Rial J, Salas A *et al*: **Development and Validation of a New Clinical Scale for Infants with Acute Respiratory Infection: The ReSVinet Scale**. *PLoS One* 2016, **11**(6):e0157665.

18. Iroh Tam PY, Sadoh AE, Obaro SK: **A meta-analysis of antimicrobial susceptibility profiles for pneumococcal pneumonia in sub-Saharan Africa**. *Paediatr Int Child Health* 2018, **38**(1):7-15.

19. Iroh Tam PY: **The Challenge and Opportunity of Pediatric Antimicrobial Stewardship in Low Resource Settings**. *J Trop Pediatr* 2020, **66**(1):1-3.

For peer review only - http://bmjopen.bmj.com/site/about/guidelines.xhtml

Table 1. List of variables used to compute RISC scores

RISC	Score
Oxygen saturation ≤90%	3
Chest indrawing	2
Refusal to feed	1
Wheezing	-2
WHO weight for age z-score ≤ -3	2
-3 ≤_z < -2	1

RISC, Respiratory Index of Severity in Children

For peer review only

Table 2. Characteristics of children hospitalised with clinical pneumonia, stratified by HIV exposure status

Characteristics	All (N=1139)	Group: HEU (N=101)	Group: HUU (N=1038)	p-value
**Demographic and physiological characteristics**				
Age, months (m)				0.02†
N	1118	99	1019	
Median (IQR)	11.0 (6.0, 20.0)	9.0 (5.0, 15.5)	11.0 (6.0, 20.0)	
Age group				
N	1116	99	1017	
0-2 m	126 (11.3)	13 (13.1)	113 (11.1)	
2-6 m	188 (16.9)	23 (23.2)	165 (16.2)	
6-12 m	302 (27.1)	32 (32.3)	270 (26.6)	
12-24 m	318 (28.5)	18 (18.2)	300 (29.5)	
24-60 m	182 (16.3)	13 (13.1)	169 (16.6)	
Sex				0.92*
N	1139	101	1038	
Male (%)	677 (59.4)	61 (60.4)	616 (59.3)	
Birth weight, kg				0.03†
N	1117	97	1020	
Median (IQR)	3.0 (2.3, 3.3)	2.8 (1.8, 3.2)	3.0 (2.4, 3.3)	
Low birth weight				0.04*
N	1117	97	1020	
< 2.5 kg (%)	302 (27.0)	35 (36.1)	267 (26.2)	
Weight for height				< 0.01¥
N	1098	100	998	
Mean (SD)	-0.7 (2.5)	-1.2 (2.5)	-0.6 (2.5)	
Weight for height categories				
N	1098	101	998	
<-3 (%)	106 (9.7)	15 (15. 0)	91 (9.1)	
-3- -2 (%)	248 (0.9)	31 (31.0)	217 (21.7)	
>-2 (%)	1102 (98.6)	54 (54.0)	690 (69.1)	
MUAC, cm				< 0.01†
N	1129	101	1028	
Median (IQR)	14.0 (13.0, 15. 0)	13.6 (12.6, 14.6)	14.0 (13.0, 15.0)	
MUAC categories				
N	1139	101	1038	
< 11.5 cm (%)	47 (4.1)	13 (12.9)	34 (3.3)	

For peer review only

For peer review only - http://bmjopen.bmj.com/site/about/guidelines.xhtml

Previous visit to health centre				1.00*
N	1139	101	1038	
Yes (%)	979 (86.0)	87 (86.1)	892 (85.9)	
Temperature				0.02ǂ
N	1133	100	1033	
Median (IQR)	37.8 (36.9, 38.6)	38.0 (37.2, 39.0)	37.8 (36.8, 38.5)	
Fever				0.06
N	1133	100	1033	
Yes (%)	518 (45.7)	55 (55.0)	463 (44.8)	
Antibiotic use				0.34*
N	1131	101	1030	
Yes (%)	451 (39.9)	45 (44.6)	406 (39.4)	
Inability to drink				0.08*
N	1134	101	1033	
Yes (%)	91 (8.0)	13 (12.9)	78 (7.6)	
Vomiting				0.02*
N	1137	101	1036	
Yes (%)	261 (23.0)	33 (32.7)	228 (22.0)	
At least one IMCI danger sign				0.05*
N	1139	101	1038	
Yes (%)	424 (37.2)	47 (46.5)	377 (36.3)	
**Household characteristics**				
Household size >4				0.83*
N	1136	101	1035	
Yes (%)	480 (42.3)	44 (43.6)	436 (42.1)	
**Clinical features**				
Nasal flaring				0.92*
N	1137	101	1036	
Yes (%)	608 (53.5)	55 (54.5)	553 (53.4)	
Lower chest wall indrawing				1.00*
N	1137	101	1036	
Yes (%)	605 (53.2)	54 (53.5)	551 (53.2)	
Stridor				0.44*
N	1137	101	1036	
Yes (%)	22 (1.9)	3 (3.0)	19 (1.8)	
Crackles				0.75*
N	1139	101	1038	
Yes (%)	623 (54.7)	57 (56.4)	566 (54.5)	
Wheezing				0.41*
N	1137	101	1036	
Yes (%)	192 (16.9)	20 (19.8)	172 (16.6)	

For peer review only - http://bmjopen.bmj.com/site/about/guidelines.xhtml

For peer review only

Bronchial breathing				0.12*
N	1139	101	1038	
Yes (%)	70 (6.2)	10 (9.9)	60 (5.8)	
Reduced air entry				0.19*
N	1139	101	1038	
Yes	9 (0.8)	2 (2.0)	7 (0.7)	
Age-adjusted tachycardia				0.11*
N	505	46	459	
Age 0-12m and >160 beats/min	266 (52.7)	31 (67.4)	235 (51.2)	
Age 12-36m and >150 beats/min	204 (40.4)	14 (30.4)	190 (41.4)	
Age 36-60m and >140 beats/min	35 (6.9)	1 (2.2)	34 (7.4)	
Age-defined tachypnoea				0.02*
N	666	57	609	
Age 0-12m and >50 breaths/min	342 (51.4)	39 (68.4)	303 (49.8)	
Age 12-60m and >40 breaths/min	324 (48.7)	18 (31.6)	306 (50.3)	
Oral thrush/sores				0.11*
N	1138	101	1037	
Yes (%)	21 (1.9)	4 (4.0)	17 (1.6)	
Hepatomegaly				0.61*
N	1135	99	1036	
Yes (%)	11 (1.0)	0 (0.0)	11 (1.1)	
Oxygen saturation				0.28†
N	1128	100	1028	
Median (IQR)	95.0 (89.0, 98.0)	95.0 (86.0, 98.0)	95.0 (89.8, 98.0)	
Hypoxemia <90%				0.40*
N	1128	100	1028	
Yes (%)	286 (25.4)	29 (29.0)	257 (25.0)	
**Risk scores**				
RISC				<0.01*
N	1120	100	1020	
-2,-3	1004 (89.6)	80 (80.0)	924 (90.6)	
3-6	116 (10.4)	20 (20.0)	96 (9.4)	
**Clinical management**				
Received oxygen				0.04*
N	1129	100	1029	
Yes (%)	383 (33.9)	44 (44.0)	339 (32.9)	
Placed on CPAP				0.14*
N	1118	99	1019	

For peer review only - http://bmjopen.bmj.com/site/about/guidelines.xhtml

Yes (%)	23 (2.1)	4 (4.0)	19 (1.9)	
Received IVF				1.00*
N	1120	100	1020	
Yes (%)	14 (1.3)	1 (1.0)	13 (1.3)	
**Outcomes**				
Length of hospital stay, days				0.21†
N	1086	96	990	
Median (IQR)	2.0 (2.0, 4.0)	2.0 (2.0, 4.0)	2.0 (2.0, 3.0)	
Mortality				0.07*
N	1139	101	1038	
Yes (%)	5 (0.4)	2 (2.0)	3 (0.3)	

CPAP, continuous positive airway pressure; HEU, HIV-exposed, uninfected; HUU, HIV-unexposed, uninfected; IMCI, Integrated Management of Childhood Illness; IQR, interquartile range; IVF, intravenous fluids; NA, not applicable; MUAC, mid-upper arm circumference; RISC, Respiratory Index of Severity in Children

** Fisher's exact test*

† Wilcoxon rank sum test

¥ Independent two sample t-test

For peer review only

For peer review only - http://bmjopen.bmj.com/site/about/guidelines.xhtml

Figure legend

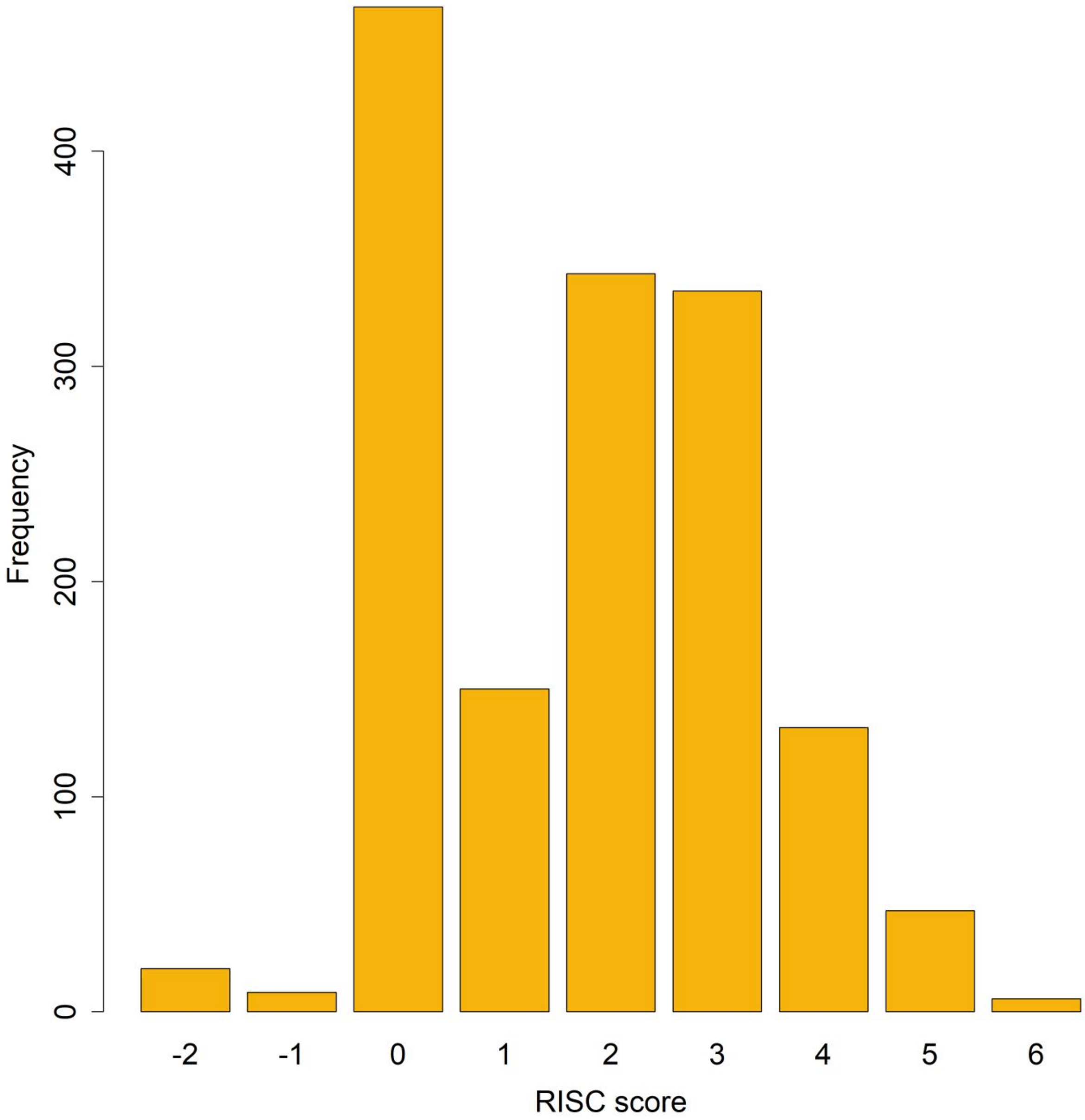

Fig 1. Summary of RISC scores computed from the data

RISC, Respiratory Index of Severity in Children

BMJ Open

Clinical pneumonia in the hospitalised child in Malawi in the post-pneumococcal conjugate vaccine era: a prospective hospital-based observational study

Journal:	*BMJ Open*
Manuscript ID	bmjopen-2021-050188.R1
Article Type:	Original research
Date Submitted by the Author:	01-Sep-2021
Complete List of Authors:	Iroh Tam, Pui-Ying ; Malawi-Liverpool-Wellcome Trust Clinical Research Programme, Department of Paediatrics and Child Health; Liverpool School of Tropical Medicine, Department of Paediatrics and Child Health<br>Chirombo, James; Malawi-Liverpool-Wellcome Trust Clinical Research Programme<br>Henrion, Marc; Malawi-Liverpool-Wellcome Trust Clinical Research Programme; Liverpool School of Tropical Medicine,<br>Newberry, Laura; Malawi-Liverpool-Wellcome Trust Clinical Research Programme, Department of Paediatrics and Child Health<br>Mambule, Ivan; Malawi-Liverpool-Wellcome Trust Clinical Research Programme<br>Everett, Dean; Malawi-Liverpool-Wellcome Trust Clinical Research Programme, Department of Paediatrics and Child Health; Khalifa University<br>Mwansambo, Charles; Government of Malawi Ministry of Health<br>Cunliffe, Nigel; University of Liverpool, Clinical Infection, Microbiology and Immunology<br>French, Neil; University of Liverpool Faculty of Health and Life Sciences, Centre for Global Vaccine Research, Institute of Infection and Global Health<br>Heyderman, Robert; University College London, Division of Infection and Immunity<br>Bar-Zeev, Naor ; Johns Hopkins University Bloomberg School of Public Health, Global Disease Epidemiology and Contro
**Primary Subject Heading**:	Paediatrics
Secondary Subject Heading:	Global health, HIV/AIDS, Respiratory medicine
Keywords:	Epidemiology < INFECTIOUS DISEASES, Paediatric infectious disease & immunisation < PAEDIATRICS, Respiratory infections < THORACIC MEDICINE

SCHOLARONE™
Manuscripts

I, the Submitting Author has the right to grant and does grant on behalf of all authors of the Work (as defined in the below author licence), an exclusive licence and/or a non-exclusive licence for contributions from authors who are: i) UK Crown employees; ii) where BMJ has agreed a CC-BY licence shall apply, and/or iii) in accordance with the terms applicable for US Federal Government officers or employees acting as part of their official duties; on a worldwide, perpetual, irrevocable, royalty-free basis to BMJ Publishing Group Ltd ("BMJ") its licensees and where the relevant Journal is co-owned by BMJ to the co-owners of the Journal, to publish the Work in this journal and any other BMJ products and to exploit all rights, as set out in our licence.

The Submitting Author accepts and understands that any supply made under these terms is made by BMJ to the Submitting Author unless you are acting as an employee on behalf of your employer or a postgraduate student of an affiliated institution which is paying any applicable article publishing charge ("APC") for Open Access articles. Where the Submitting Author wishes to make the Work available on an Open Access basis (and intends to pay the relevant APC), the terms of reuse of such Open Access shall be governed by a Creative Commons licence – details of these licences and which Creative Commons licence will apply to this Work are set out in our licence referred to above.

Other than as permitted in any relevant BMJ Author's Self Archiving Policies, I confirm this Work has not been accepted for publication elsewhere, is not being considered for publication elsewhere and does not duplicate material already published. I confirm all authors consent to publication of this Work and authorise the granting of this licence.

For peer review only

Clinical pneumonia in the hospitalised child in Malawi in the post-pneumococcal conjugate vaccine era: a prospective hospital-based observational study

Pui-Ying Iroh Tam,[1,2,3,a*] James Chirombo,[4*] Marc Y. R. Henrion,[3,4] Laura Newberry,[1,2,b] Ivan Mambule,[1,5,c] Dean Everett,[1,5,d] Charles Mwansambo,[6] Nigel A. Cunliffe,[5] Neil French,[5] Robert S. Heyderman,[7] and Naor Bar-Zeev[1,2,5,e] for the VacSurv Consortium

[1]Paediatrics and Child Health Research Group, Malawi-Liverpool-Wellcome Trust Clinical Research Programme, Blantyre, Malawi

[2]Department of Paediatrics and Child Health, University of Malawi College of Medicine, Blantyre, Malawi

[3]Department of Clinical Sciences, Liverpool School of Tropical Medicine, Liverpool, UK

[4]Statistical Support Unit, Malawi-Liverpool-Wellcome Trust Clinical Research Programme, Blantyre, Malawi

[5]Institute of Infection, Veterinary and Ecological Science, University of Liverpool, Liverpool, UK

[6]Malawi Ministry of Health, Lilongwe, Malawi

[7]Research Department of Infection, Division of Infection & Immunity, University College London, London, UK

*These authors contributed equally to the manuscript.

[a]Corresponding author: irohtam@mlw.mw

For peer review only - http://bmjopen.bmj.com/site/about/guidelines.xhtml

For peer review only

[b]Current address: Kaiser Permanente, Parker, CO, USA

[c]Current address: Medical Research Council, Kampala, Uganda

[d]Current address: Khalifa University, Abu Dhabi, UAE

[e]Current address: Johns Hopkins School of Public Health, Baltimore, MD, USA

Short title: Clinical pneumonia in the Malawian child

<u>Abbreviations</u>

CI, confidence interval

CPAP, continuous positive airway pressure

HEU, HIV-exposed, uninfected

HUU, HIV-unexposed, uninfected

IMCI, integrated management of childhood illness

IQR, interquartile range

mRISC, modified respiratory index of severity in children

MUAC, mid-upper arm circumference

PCV13, 13-valent pneumococcal conjugate vaccine

ResViNet, Respiratory Syncytial Virus Network

RISC, respiratory index of severity in children

For peer review only

Abstract

Objective

Assess characteristics of clinical pneumonia after introduction of pneumococcal conjugate vaccine (PCV), by HIV exposure status, in children hospitalized in a governmental hospital in Malawi.

Methods and findings

We evaluated 1,139 children ≤5 years old hospitalised with clinical pneumonia: 101 HIV-exposed uninfected (HEU) and 1038 HIV-unexposed, uninfected (HUU). Median age was 11 months (IQR 6-20), 59% were male, median mid-upper arm circumference (MUAC) was 14 cm (IQR 13-15) and mean weight-for-height z score was -0.7 (±2.5). The highest Respiratory Index of Severity in Children (RISC) scores were allocated to 10.4% of the overall cohort, respectively. Only 45.7% had fever, and 37.2% had at least one danger sign at presentation. The most common clinical features were crackles (54.7%), nasal flaring (53.5%), and lower chest wall indrawing (53.2%). Compared to HUU, HEU children were significantly younger (9 months v. 11 months), with lower mean birth weight (2.8 kg v. 3.0 kg) and MUAC (13.6 cm v. 14.0 cm), had higher prevalence of vomiting (32.7% v. 22.0%), tachypnoea (68.4% v. 49.8%), and highest RISC scores (20.0% v. 9.4%). Five children died (0.4%). However, clinical outcomes were similar for both groups.

Conclusions

For peer review only

In this post-PCV setting where prevalence of HIV and malnutrition is high, children hospitalised fulfilling the WHO Integrated Management of Childhood Illness criteria for clinical pneumonia present with heterogeneous features. These vary by HIV exposure status but this does not influence either the frequency of danger signs or mortality. The poor performance of available severity scores in this population and the absence of more specific diagnostics hinder appropriate antimicrobial stewardship and the rational application of other interventions.

266 words (max 250)

Keywords: children, HIV-exposed, pneumonia, predictive value, respiratory tract, risk score

For peer review only

Strengths and limitations of the study

- We evaluated over 1,100 children hospitalized with pneumonia in a low-income country setting after introduction of PCV.
- This observational cohort was nested within a prospective hospital-based study of PCV13 effectiveness
- We assessed the demographic and clinical characteristics of clinical pneumonia patients and compared HEU vs. HUU children, and computed RISC scores for severe pneumonia

For peer review only

For peer review only - http://bmjopen.bmj.com/site/about/guidelines.xhtml

Introduction

Pneumonia is the leading cause of morbidity and mortality in children globally. This disease is of particular importance in low-income countries such as Malawi, where HIV prevalence among pregnant women is 10.8% [1], and where a high burden of malaria and malnutrition (stunting prevalence of 39% [2]) contributes to an under-5 mortality rate of 55 per 1,000 live births [3].

WHO Integrated Management of Childhood Illness (IMCI) guidelines for community and referral level management of children with pneumonia rely upon clinical recognition of features of severe disease. WHO lists general danger signs, such as inability to drink, persistent vomiting, convulsions, lethargy or unconsciousness, or stridor in a calm child, which are clinical features used to identify children who should be treated in an inpatient setting and to receive injectable antibiotics [4]. However, clinical features are non-specific and can reflect a variety of other infectious (e.g. bronchiolitis, malaria, sepsis, tuberculosis, pneumocystis), and non-infectious (e.g. asthma, lymphocytic interstitial pneumonitis, pulmonary Kaposi sarcoma) conditions. Inappropriate and overuse of antimicrobials in low resource settings where antimicrobial prescribing is based more on clinical features than objective diagnostic data has implications for antimicrobial stewardship in the post-pneumococcal conjugate vaccine (PCV) era, particularly in settings where antimicrobial choice and supply is limited and antimicrobial resistance rates are rising [5].

Few risk scoring systems to predict severe pneumonia exist that are adaptable to low-income countries with high HIV prevalence – Respiratory Index of Severity in Children-Malawi (RISC-

For peer review only - http://bmjopen.bmj.com/site/about/guidelines.xhtml

Malawi) is the only score from children from a low-income country, and did not consider HIV status. Furthermore, although the alternative, Respiratory Index of Severity in Children (RISC) score does include a scoring system based on HIV infection, no studies have specifically evaluated whether HIV exposure status affects the presence and predictive value of clinical features for severe disease and case-fatality. As HIV-exposed, uninfected (HEU) children constitute a growing population and, compared to HIV-unexposed, uninfected (HUU) remain vulnerable to more severe pneumonia, more often fail empiric pneumonia treatment, and have higher rates of hospitalisation and death than HIV-unexposed, uninfected (HUU) children [6-9], this population is of growing clinical relevance. Our objectives were to describe the clinical features of children hospitalised with pneumonia, calculate risk scores for our population, and to determine whether differences in clinical characteristics and calculated risk scores exist by HIV exposure status.

Methods

This observational cohort was nested within a prospective hospital-based study of 13-valent PCV effectiveness conducted at Queen Elizabeth Central Hospital between 2 April 2013 and 3 August 2016. Children born on or after 1 October 2012 (based on verbal and written documentation from parents on time since birth) and therefore eligible for PCV and admitted with a diagnosis of clinical pneumonia were enrolled (Supplementary Table 1). Children were excluded if they had known oncological or congenital heart disease, were admitted ≥48 hours before recruitment, were re-admitted to hospital within 14 days of a previous hospitalisation, or presented moribund with impending death. Study staff performed active case finding Monday-Friday during daytime

For peer review only - http://bmjopen.bmj.com/site/about/guidelines.xhtml

in the emergency department and inpatient wards, and followed up study participants 6 weeks following hospital discharge with a home visit or phone call to confirm vital status.

With informed written parental consent, all enrolled children had baseline characteristics measured which included clinical, laboratory and sociodemographic variables. Oxygen saturation was measured with an appropriately sized sensor and a Nellcor pulse oximeter (Welch Allyn Spot Vital Signs Devices, Skaneateles Falls, NY, USA) in room air. Children with oxygen saturations <90% in room air were given supplemental oxygen via nasal cannula using an oxygen concentrator. At clinician decision and if a free circuit was available, children with severe respiratory distress, signs of exhaustion or worsening hypoxemia received bubble continuous positive airway pressure (Pumani, 3rd Stone Design, San Rafael, CA, USA). Blood was routinely collected for packed cell volume and malaria parasite thick film. Mothers and infants were tested for HIV according to the Malawi National HIV Guidelines [10]. A blood culture was obtained only when the admitting clinician suspected sepsis. Children with suspected meningitis, malaria or anaemia, were managed according to respective WHO and hospital treatment guidelines [11].

This study was approved by the University of Malawi College of Medicine Research Ethics Committee (P.05/13/1386) and by the University of Liverpool (RETH490).

Patient and public involvement

Patients or the public were not involved in the design, or conduct, or reporting, or dissemination plans of our research.

For peer review only - http://bmjopen.bmj.com/site/about/guidelines.xhtml

Case definition

Pneumonia was diagnosed clinically per WHO IMCI criteria.

Statistical analysis

We assessed the demographic and clinical characteristics of clinical pneumonia patients and compared HEU vs. HUU children using Fisher's exact test, two-sample t-test or Wilcoxon rank-sum tests respectively for categorical, continuous normal and continuous skewed variables.

We computed RISC scores for severe pneumonia in our cohort [12-14], using the variables listed in Table 1. We had insufficient variables to compute modified Respiratory Index of Severity in Children (mRISC) and RISC-Malawi scores [13].

Results

A total of 1660 children were recruited into the study, among whose files were incomplete for 521. We included 1,139 children in the analysis, of whom 101 were HEU and 1,038 HUU. The median age was 11 months (IQR 6.0, 20.0), 59% were male, and the median mid-upper arm circumference (MUAC) was 14 cm (IQR 13-15) and mean weight-for-height z score -0.7 (±2.5; Table 2 and Supplementary Table 2).

In our cohort, no single clinical feature was present at enrollment in more than 55% of the population: the most common clinical features were crackles (54.7%), nasal flaring (53.5%), and lower chest wall indrawing (53.2%). Less than half had fever (45.7%), and at least one IMCI

For peer review only - http://bmjopen.bmj.com/site/about/guidelines.xhtml

danger sign (37.2%). The median oxygen saturation at presentation was 95% (IQR 89,98), with 25.4% having oxygen saturations below 90%. Among this cohort, 33.9% received oxygen and 2.1% were placed on CPAP. The highest RISC scores (3+) were allocated to 10.4% of the overall cohort, respectively. Five children died (0.4%).

Ten percent of children in our cohort were HEU, and notable differences between HEU and HUU were observed in several areas. In the HEU group, children were younger (median age 9 months (IQR 5.0-15.5) v. 11 months (IQR 6.0-20.0), p=0.02), with lower birth weight (median 2.8 kg (IQR 1.8-3.2) v. 3.0 kg (IQR 2.4-3.3), p=0.03), lower weight-for-height z score (mean -1.2±2.5 vs. -0.6±2.5, p<0.01) and lower MUAC (median 13.6 cm (IQR 12.6-14.6) v. 14 cm (IQR 13-15), p<0.01). Compared to the HUU group, HEU children had significantly higher prevalence of vomiting (32.7% v. 22.0%, p=0.02) and age-defined tachypnoea in infancy (68.4% v. 49.8%, p=0.02). More HEU children presented with at least one IMCI danger sign (46.5% v. 36.3%, p=0.05). We did not observe significant differences in respiratory symptoms and in proportions of children who died between groups, although HEU children received oxygen at a significantly higher rate than HUU children (44.0% v. 32.9%, p=0.04). Clinical outcomes between the HEU and HUU groups were also similar. However, RISC scores were significantly different between HIV exposure status, with 20.0% of HEU with the highest scores, compared to 9.4% of HUU (Fig 1). The risk score had a nonparametric distribution and many subjects scored 0.

Discussion

In our prospective cohort of children presenting with WHO IMCI criteria for clinical pneumonia in the post-PCV era, the most common clinical features were crackles, nasal flaring, and lower

For peer review only - http://bmjopen.bmj.com/site/about/guidelines.xhtml

chest wall indrawing. Despite the clinical diagnosis of pneumonia, features were heterogeneous. Thirty-four percent had an oxygen requirement and 2.1% needed CPAP support, though with best available treatment, overall mortality was low – and possibly a reflection of improvements in child health overall as a result of antiretroviral and antimalarial therapies, vaccination, and community management of nutrition. Ten percent of children hospitalised with pneumonia were HEU and this group was significantly younger, with lower birth weight and anthropometric parameters. HEU children had a significantly higher prevalence of vomiting, age-defined tachypnoea in infancy, and higher RISC scores, compared to HUU children.

Though HEU children were significantly younger and had significantly lower MUAC than HUU children, and therefore age and anthropometry are potential triage indicators for use in the clinical management of pneumonia, these differences were subtle and may not be useful for a clinician, who in low resource settings make decisions primarily on clinical suspicion. WHO IMCI clinical criteria for pneumonia are non-specific, and based on presence of cough, fast and difficult breathing. The non-specific nature of the diagnosis, which covers both infectious and non-infectious causes, is deliberately intended to increase sensitivity; however, as reflected in this study, this may have resulted in the term being used with a variety of features. Much research has been expended on developing pneumonia risk scores to identify children at risk of severe disease. However, the limitations of existing pneumonia risk scores for children in low- and middle-income country settings [15], including Pneumonia Etiology Research in Child Health (PERCH) [16], Respiratory Syncytial Virus Network (ReSViNet) [17], RISC [14], mRISC [12], and RISC-Malawi [13], is that the predictive capacity is generally modest and, in the case of PERCH, was found to be not as discriminatory as the WHO danger signs. The risk

For peer review only - http://bmjopen.bmj.com/site/about/guidelines.xhtml

scores we calculated in our cohort were unevenly distributed and yielded surprisingly divergent results, indicating that they are limited to their study populations and are not generalisable.

In our cohort, the lack of predominating features of clinical pneumonia points to another possibility that, in post-PCV settings where HIV and chronic malnutrition rates are high (10% and 39%, respectively), children do not present with typical signs and symptoms. The difference in distribution of risk scores within our cohort highlights the fact that risk scores are population- and cohort-specific. In our cohort a substantial proportion of children met criteria for severe acute malnutrition (9.7% had weight-for-height z scores ≤3; and 4.1% had MUAC <11.5 cm), indicating how widespread chronic malnutrition is in Malawi. This pervasive underlying comorbidity leads to subsequent issues with identification and management of disease; and may in part explain overuse of antimicrobials in this setting, one factor contributing to an increase in antimicrobial resistance.

Antimicrobial stewardship and infection control issues in low-resource settings have been particularly problematic among young children, with significant increases in antimicrobial resistance noted among young infants with bloodstream infections over a twenty-year period [5], and concerns over resistance rates in childhood pneumonia in sub-Saharan Africa [18]. Challenges with diagnostic capacity and the limitations of clinical diagnosis hinder appropriate antimicrobial stewardship [19]. Risk scores that are adequately discriminatory therefore present an opportunity for targeted interventions, including early aggressive support, as well as judicious allocation of resources.

For peer review only - http://bmjopen.bmj.com/site/about/guidelines.xhtml

This study has several limitations. Only a very small proportion of HIV-exposed infants had HIV status tested by PCR, as recommended by national guidelines. This was due to shortage of HIV counselors, lack of testing reagents and reluctance of parents to obtain additional testing on their children. We did not recruit moribund children who died very soon after arrival, and therefore we may have underestimated our fatality rate. A substantial proportion of recruited participants had incomplete files and were therefore not included in the analysis. Due to small numbers, we were unable to calculate predictive values for severe outcomes. However, this was a detailed prospective study conducted in a low resource setting in the post-PCV era where robust diagnostic testing was available.

In conclusion, in this setting where prevalence of HIV and malnutrition is high, children hospitalised fulfilling the WHO IMCI criteria for clinical pneumonia in the post-PCV era present with heterogeneous features. These vary by HIV exposure status but this does not influence either the frequency of danger signs or mortality. The sample size limited our ability to calculate predictive scores; and the performance of available severity scores in this population and the absence of more specific diagnostics hinder appropriate antimicrobial stewardship and the rational application of other interventions. Further work in developing antimicrobial stewardship scores in resource-limited settings, in a period defined by rising rates of antimicrobial resistance, may be warranted.

1,990 words

For peer review only - http://bmjopen.bmj.com/site/about/guidelines.xhtml

297 **Acknowledgements**

298 The authors wish to thank the collaborating members of the VacSurv Consortium (James Beard,
299 Amelia C Crampin, Carina King, Sonia Lewycka, Hazzie Mvula, Tambosi Phiri, Miren Iturriza-
300 Gomara, Osamu Nakagomi, Jennifer Verani, Cynthia Whitney).

301

302 **Funding statement**

303 This work was supported by a Wellcome Trust Programme Grant (Grant number 091909/Z/10/Z)
304 and the MLW Programme Core Award (Grant 206454) from the Wellcome Trust. The funders
305 had no role in study design, data collection and analysis, decision to publish, or preparation of
306 the manuscript. The findings and conclusions in this report are those of the authors and do not
307 necessarily represent the official position of the Centers for Disease Control and Prevention,
308 Atlanta USA.

309

310 **Competing interests**

311 PI has received investigator-initiated research grant support from Bill & Melinda Gates
312 Foundation outside the scope of this work; NAC has received research grant support and
313 honoraria for participation in rotavirus vaccine advisory board meetings from GlaxoSmithKline
314 Biologicals and from Takeda Pharmaceuticals outside the scope of this work; NF has received
315 investigator-initiated research grant support from GlaxoSmithKline Biologicals and from Takeda
316 Pharmaceuticals outside the scope of this work. NBZ has received investigator-initiated research
317 grant support from GlaxoSmithKline Biologicals, Takeda Pharmaceuticals, Merck-Sharpe-
318 Dohme and the Serum Institute of India, all outside the scope of this work. All other authors
319 declare that they have no financial disclosures or competing interests.

For peer review only

Author contributorship statement

Conceived and designed the research question: PI, NBZ; Conducted the statistical analysis: JC, MH; Managed the study: LN, IM; Developed and awarded funding for the study: DE, NAC, CM, NF, RH; Wrote the first draft of the manuscript: PI; Provided critical feedback on the manuscript: JC, MH, NAC, NF, RH, NBZ; Reviewed and approved the final draft: all authors.

Data availability

Data is available upon reasonable request to the corresponding author.

Ethics statement

This study was approved by the University of Malawi College of Medicine Research Ethics Committee (P.05/13/1386) and by the University of Liverpool (RETH490).

For peer review only

References

1. UNAIDS: **Malawi factsheet**. 2019.

2. World Bank: **Prevalence of stunting - Malawi**. 2018.

3. WHO: **Malawi Maternal and Child Health Data**. Geneva, Switzerland: WHO; 2015.

4. WHO: **Revised WHO classification and treatment of childhood pneumonia at health facilities**. Geneva, Switzerland: WHO; 2014.

5. Iroh Tam PY, Musicha P, Kawaza K, Cornick J, Denis B, Freyne B, Everett D, Dube Q, French N, Feasey N *et al*: **Emerging Resistance to Empiric Antimicrobial Regimens for Pediatric Bloodstream Infections in Malawi (1998-2017)**. *Clin Infect Dis* 2019, **69**(1):61-68.

6. Landes M, van Lettow M, Chan AK, Mayuni I, Schouten EJ, Bedell RA: **Mortality and health outcomes of HIV-exposed and unexposed children in a PMTCT cohort in Malawi**. *PLoS One* 2012, **7**(10):e47337.

7. Slogrove A, Reikie B, Naidoo S, De Beer C, Ho K, Cotton M, Bettinger J, Speert D, Esser M, Kollmann T: **HIV-exposed uninfected infants are at increased risk for severe infections in the first year of life**. *J Trop Pediatr* 2012, **58**(6):505-508.

8. Slogrove AL, Goetghebuer T, Cotton MF, Singer J, Bettinger JA: **Pattern of Infectious Morbidity in HIV-Exposed Uninfected Infants and Children**. *Front Immunol* 2016, **7**:164.

9. Zar HJ, Barnett W, Stadler A, Gardner-Lubbe S, Myer L, Nicol MP: **Aetiology of childhood pneumonia in a well vaccinated South African birth cohort: a nested**

For peer review only

case-control study of the Drakenstein Child Health Study. *Lancet Respir Med* 2016, **4**(6):463-472.

10. Malawi Ministry of Health: **Clinical Management of HIV in Adults and Children**. Malawi; 2011.

11. WHO: **Handbook IMCI. Integrated management of childhood illness**. In. Geneva, Switzerland: World Health Organization; 2000.

12. Emukule GO, McMorrow M, Ulloa C, Khagayi S, Njuguna HN, Burton D, Montgomery JM, Muthoka P, Katz MA, Breiman RF *et al*: **Predicting mortality among hospitalized children with respiratory illness in Western Kenya, 2009-2012**. *PLoS One* 2014, **9**(3):e92968.

13. Hooli S, Colbourn T, Lufesi N, Costello A, Nambiar B, Thammasitboon S, Makwenda C, Mwansambo C, McCollum ED, King C: **Predicting Hospitalised Paediatric Pneumonia Mortality Risk: An External Validation of RISC and mRISC, and Local Tool Development (RISC-Malawi) from Malawi**. *PLoS One* 2016, **11**(12):e0168126.

14. Reed C, Madhi SA, Klugman KP, Kuwanda L, Ortiz JR, Finelli L, Fry AM: **Development of the Respiratory Index of Severity in Children (RISC) score among young children with respiratory infections in South Africa**. *PLoS One* 2012, **7**(1):e27793.

15. Deardorff KV, McCollum ED, Ginsburg AS: **Pneumonia Risk Stratification Scores for Children in Low-Resource Settings: A Systematic Literature Review**. *Pediatr Infect Dis J* 2018, **37**(8):743-748.

16. Gallagher KE, Knoll MD, Prosperi C, Baggett HC, Brooks WA, Feikin DR, Hammitt LL, Howie SRC, Kotloff KL, Levine OS *et al*: **The Predictive Performance of a**

For peer review only

For peer review only - http://bmjopen.bmj.com/site/about/guidelines.xhtml

Pneumonia Severity Score in Human Immunodeficiency Virus-negative Children Presenting to Hospital in 7 Low- and Middle-income Countries. *Clin Infect Dis* 2020, **70**(6):1050-1057.

17. Justicia-Grande AJ, Pardo-Seco J, Cebey-Lopez M, Vilanova-Trillo L, Gomez-Carballa A, Rivero-Calle I, Puente-Puig M, Curros-Novo C, Gomez-Rial J, Salas A *et al*: **Development and Validation of a New Clinical Scale for Infants with Acute Respiratory Infection: The ReSVinet Scale**. *PLoS One* 2016, **11**(6):e0157665.

18. Iroh Tam PY, Sadoh AE, Obaro SK: **A meta-analysis of antimicrobial susceptibility profiles for pneumococcal pneumonia in sub-Saharan Africa**. *Paediatr Int Child Health* 2018, **38**(1):7-15.

19. Iroh Tam PY: **The Challenge and Opportunity of Pediatric Antimicrobial Stewardship in Low Resource Settings**. *J Trop Pediatr* 2020, **66**(1):1-3.

For peer review only

Table 1. List of variables used to compute RISC scores

**RISC**	**Score**
Oxygen saturation ≤90%	3
Chest indrawing	2
Refusal to feed	1
Wheezing	-2
WHO weight for age z-score ≤ -3	2
WHO weight for age z-score -3 ≤_z < -2	1

RISC, Respiratory Index of Severity in Children

For peer review only

Table 2. Characteristics of children hospitalised with clinical pneumonia, stratified by HIV exposure status

Characteristics	All (N=1139)	Group: HEU (N=101)	Group: HUU (N=1038)	p-value
**Demographic and physiological characteristics**				
Age, months (m)				0.02†
N	1118	99	1019	
Median (IQR)	11.0 (6.0, 20.0)	9.0 (5.0, 15.5)	11.0 (6.0, 20.0)	
Sex				0.92*
N	1139	101	1038	
Male (%)	677 (59.4)	61 (60.4)	616 (59.3)	
Birth weight, kg				0.03†
N	1117	97	1020	
Median (IQR)	3.0 (2.3, 3.3)	2.8 (1.8, 3.2)	3.0 (2.4, 3.3)	
Weight for height				< 0.01¥
N	1098	100	998	
Mean (SD)	-0.7 (2.5)	-1.2 (2.5)	-0.6 (2.5)	
MUAC, cm				< 0.01†
N	1129	101	1028	
Median (IQR)	14.0 (13.0, 15.0)	13.6 (12.6, 14.6)	14.0 (13.0, 15.0)	
Temperature				0.02†
N	1133	100	1033	
Median (IQR)	37.8 (36.9, 38.6)	38.0 (37.2, 39.0)	37.8 (36.8, 38.5)	
Fever				0.06
N	1133	100	1033	
Yes (%)	518 (45.7)	55 (55.0)	463 (44.8)	
Antibiotic use				0.34*
N	1131	101	1030	
Yes (%)	451 (39.9)	45 (44.6)	406 (39.4)	
Inability to drink				0.08*
N	1134	101	1033	
Yes (%)	91 (8.0)	13 (12.9)	78 (7.6)	
Vomiting				0.02*
N	1137	101	1036	
Yes (%)	261 (23.0)	33 (32.7)	228 (22.0)	
At least one IMCI danger sign				0.05*
N	1139	101	1038	
Yes (%)	424 (37.2)	47 (46.5)	377 (36.3)	

For peer review only

For peer review only - http://bmjopen.bmj.com/site/about/guidelines.xhtml

**Clinical features**				
Nasal flaring				0.92*
N	1137	101	1036	
Yes (%)	608 (53.5)	55 (54.5)	553 (53.4)	
Lower chest wall indrawing				1.00*
N	1137	101	1036	
Yes (%)	605 (53.2)	54 (53.5)	551 (53.2)	
Stridor				0.44*
N	1137	101	1036	
Yes (%)	22 (1.9)	3 (3.0)	19 (1.8)	
Age-defined tachypnoea				0.02*
N	666	57	609	
Age 0-12m and >50 breaths/min	342 (51.4)	39 (68.4)	303 (49.8)	
Age 12-60m and >40 breaths/min	324 (48.7)	18 (31.6)	306 (50.3)	
Oral thrush/sores				0.11*
N	1138	101	1037	
Yes (%)	21 (1.9)	4 (4.0)	17 (1.6)	
Hypoxemia <90%				0.40*
N	1128	100	1028	
Yes (%)	286 (25.4)	29 (29.0)	257 (25.0)	
**Risk scores**				
RISC				<0.01*
N	1120	100	1020	
-2,-3	1004 (89.6)	80 (80.0)	924 (90.6)	
3-6	116 (10.4)	20 (20.0)	96 (9.4)	
**Clinical management**				
Received oxygen				0.04*
N	1129	100	1029	
Yes (%)	383 (33.9)	44 (44.0)	339 (32.9)	
Placed on CPAP				0.14*
N	1118	99	1019	
Yes (%)	23 (2.1)	4 (4.0)	19 (1.9)	
**Outcomes**				
Length of hospital stay, days				0.21‡
N	1086	96	990	
Median (IQR)	2.0 (2.0, 4.0)	2.0 (2.0, 4.0)	2.0 (2.0, 3.0)	
Mortality				0.07*
N	1139	101	1038	
Yes (%)	5 (0.4)	2 (2.0)	3 (0.3)	

For peer review only

CPAP, continuous positive airway pressure; HEU, HIV-exposed, uninfected; HUU, HIV-unexposed, uninfected; IMCI, Integrated Management of Childhood Illness; IQR, interquartile range; MUAC, mid-upper arm circumference; RISC, Respiratory Index of Severity in Children

** Fisher's exact test*

† Wilcoxon rank sum test

¥ Independent two sample t-test

For peer review only

Figure legend

Fig 1. Summary of RISC scores computed from the data

RISC, Respiratory Index of Severity in Children

Appendix

Supplementary Table 1. STROBE checklist

Supplementary Table 2. Full characteristics of children hospitalised with clinical pneumonia, stratified by HIV exposure status

For peer review only

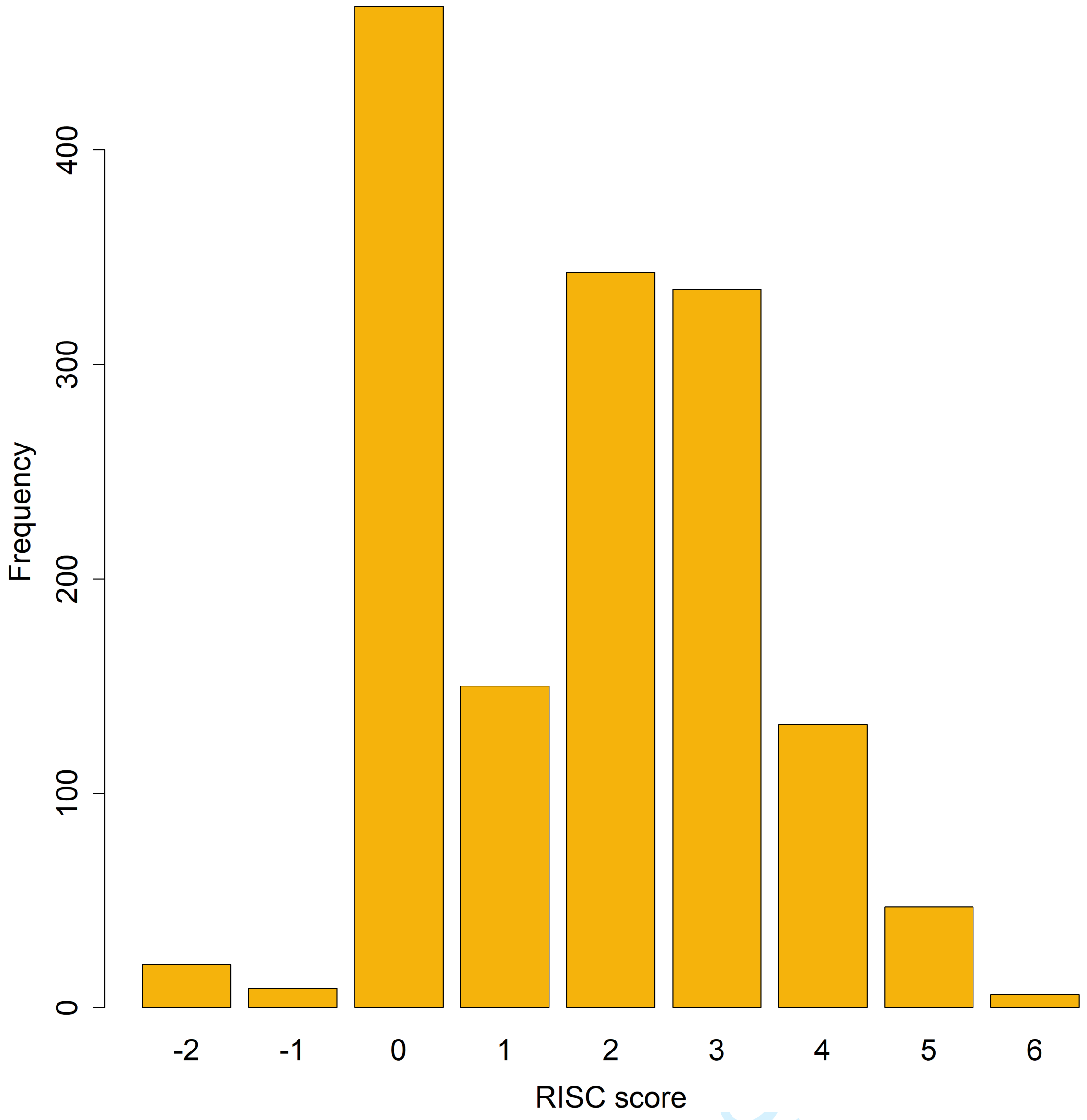
Frequency
0
100
200
300
400
-2
-1
0
1
2
3
4
5
6
RISC score

Supplementary Table 1. STROBE Statement—Checklist of items that should be included in reports of ***cohort studies***

	Item No	Recommendation	Page
**Title and abstract**	1	(*a*) Indicate the study's design with a commonly used term in the title or the abstract	1
		(*b*) Provide in the abstract an informative and balanced summary of what was done and what was found	3
**Introduction**			
Background/rationale	2	Explain the scientific background and rationale for the investigation being reported	6
Objectives	3	State specific objectives, including any prespecified hypotheses	7
**Methods**			
Study design	4	Present key elements of study design early in the paper	7
Setting	5	Describe the setting, locations, and relevant dates, including periods of recruitment, exposure, follow-up, and data collection	7
Participants	6	(*a*) Give the eligibility criteria, and the sources and methods of selection of participants. Describe methods of follow-up	7
		(*b*) For matched studies, give matching criteria and number of exposed and unexposed	N/A
Variables	7	Clearly define all outcomes, exposures, predictors, potential confounders, and effect modifiers. Give diagnostic criteria, if applicable	8
Data sources/ measurement	8*	For each variable of interest, give sources of data and details of methods of assessment (measurement). Describe comparability of assessment methods if there is more than one group	8
Bias	9	Describe any efforts to address potential sources of bias	9
Study size	10	Explain how the study size was arrived at	9
Quantitative variables	11	Explain how quantitative variables were handled in the analyses. If applicable, describe which groupings were chosen and why	9
Statistical methods	12	(*a*) Describe all statistical methods, including those used to control for confounding	9
		(*b*) Describe any methods used to examine subgroups and interactions	9
		(*c*) Explain how missing data were addressed	9
		(*d*) If applicable, explain how loss to follow-up was addressed	9
		(*e*) Describe any sensitivity analyses	N/A
**Results**			
Participants	13*	(a) Report numbers of individuals at each stage of study—eg numbers potentially eligible, examined for eligibility, confirmed eligible, included in the study, completing follow-up, and analysed	9
		(b) Give reasons for non-participation at each stage	9
		(c) Consider use of a flow diagram	N/A

For peer review only - http://bmjopen.bmj.com/site/about/guidelines.xhtml

Descriptive data	14*	(a) Give characteristics of study participants (eg demographic, clinical, social) and information on exposures and potential confounders	Table 2
		(b) Indicate number of participants with missing data for each variable of interest	9
		(c) Summarise follow-up time (eg, average and total amount)	9
Outcome data	15*	Report numbers of outcome events or summary measures over time	9
Main results	16	(*a*) Give unadjusted estimates and, if applicable, confounder-adjusted estimates and their precision (eg, 95% confidence interval). Make clear which confounders were adjusted for and why they were included	9-10
		(*b*) Report category boundaries when continuous variables were categorized	Table 2
		(*c*) If relevant, consider translating estimates of relative risk into absolute risk for a meaningful time period	N/A
Other analyses	17	Report other analyses done—eg analyses of subgroups and interactions, and sensitivity analyses	N/A
**Discussion**			
Key results	18	Summarise key results with reference to study objectives	10-11
Limitations	19	Discuss limitations of the study, taking into account sources of potential bias or imprecision. Discuss both direction and magnitude of any potential bias	13
Interpretation	20	Give a cautious overall interpretation of results considering objectives, limitations, multiplicity of analyses, results from similar studies, and other relevant evidence	13
Generalisability	21	Discuss the generalisability (external validity) of the study results	13
**Other information**			
Funding	22	Give the source of funding and the role of the funders for the present study and, if applicable, for the original study on which the present article is based	14

*Give information separately for exposed and unexposed groups.

Note: An Explanation and Elaboration article discusses each checklist item and gives methodological background and published examples of transparent reporting. The STROBE checklist is best used in conjunction with this article (freely available on the Web sites of PLoS Medicine at http://www.plosmedicine.org/, Annals of Internal Medicine at http://www.annals.org/, and Epidemiology at http://www.epidem.com/). Information on the STROBE Initiative is available at http://www.strobe-statement.org.

For peer review only - http://bmjopen.bmj.com/site/about/guidelines.xhtml

Supplementary Table 2. Full characteristics of children hospitalised with clinical pneumonia, stratified by HIV exposure status

		Group		
Characteristics	All (N=1139)	HEU (N=101)	HUU (N=1038)	p-value
**Demographic and physiological characteristics**				
Age group				
N	1116	99	1017	
0-2 m	126 (11.3)	13 (13.1)	113 (11.1)	
2-6 m	188 (16.9)	23 (23.2)	165 (16.2)	
6-12 m	302 (27.1)	32 (32.3)	270 (26.6)	
12-24 m	318 (28.5)	18 (18.2)	300 (29.5)	
24-60 m	182 (16.3)	13 (13.1)	169 (16.6)	
Low birth weight				0.04*
N	1117	97	1020	
< 2.5 kg (%)	302 (27.0)	35 (36.1)	267 (26.2)	
Weight for height categories				
N	1098	101	998	
<-3 (%)	106 (9.7)	15 (15. 0)	91 (9.1)	
-3- -2 (%)	248 (0.9)	31 (31.0)	217 (21.7)	
>-2 (%)	1102 (98.6)	54 (54.0)	690 (69.1)	
MUAC categories				
N	1139	101	1038	
< 11.5 cm (%)	47 (4.1)	13 (12.9)	34 (3.3)	
Previous visit to health centre				1.00*
N	1139	101	1038	
Yes (%)	979 (86.0)	87 (86.1)	892 (85.9)	
**Household characteristics**				
Household size >4				0.83*
N	1136	101	1035	
Yes (%)	480 (42.3)	44 (43.6)	436 (42.1)	
**Clinical features**				
Crackles				0.75*
N	1139	101	1038	
Yes (%)	623 (54.7)	57 (56.4)	566 (54.5)	
Wheezing				0.41*
N	1137	101	1036	
Yes (%)	192 (16.9)	20 (19.8)	172 (16.6)	
Bronchial breathing				0.12*
N	1139	101	1038	
Yes (%)	70 (6.2)	10 (9.9)	60 (5.8)	
Reduced air entry				0.19*
N	1139	101	1038	

For peer review only

Yes	9 (0.8)	2 (2.0)	7 (0.7)	
Age-adjusted tachycardia				0.11*
N	505	46	459	
Age 0-12m and >160 beats/min	266 (52.7)	31 (67.4)	235 (51.2)	
Age 12-36m and >150 beats/min	204 (40.4)	14 (30.4)	190 (41.4)	
Age 36-60m and >140 beats/min	35 (6.9)	1 (2.2)	34 (7.4)	
Hepatomegaly				0.61*
N	1135	99	1036	
Yes (%)	11 (1.0)	0 (0.0)	11 (1.1)	
Oxygen saturation				0.28†
N	1128	100	1028	
Median (IQR)	95.0 (89.0, 98.0)	95.0 (86.0, 98.0)	95.0 (89.8, 98.0)	
**Clinical management**				
Received IVF				1.00*
N	1120	100	1020	
Yes (%)	14 (1.3)	1 (1.0)	13 (1.3)	

HEU, HIV-exposed, uninfected; HUU, HIV-unexposed, uninfected; IQR, interquartile range;

IVF, intravenous fluids; MUAC, mid-upper arm circumference

* *Fisher's exact test*

† Wilcoxon rank sum test

For peer review only

BMJ Open

Clinical pneumonia in the hospitalised child in Malawi in the post-pneumococcal conjugate vaccine era: a prospective hospital-based observational study

Journal:	*BMJ Open*
Manuscript ID	bmjopen-2021-050188.R2
Article Type:	Original research
Date Submitted by the Author:	07-Oct-2021
Complete List of Authors:	Iroh Tam, Pui-Ying ; Malawi-Liverpool-Wellcome Trust Clinical Research Programme, Department of Paediatrics and Child Health; Liverpool School of Tropical Medicine, Department of Paediatrics and Child Health<br>Chirombo, James; Malawi-Liverpool-Wellcome Trust Clinical Research Programme<br>Henrion, Marc; Malawi-Liverpool-Wellcome Trust Clinical Research Programme; Liverpool School of Tropical Medicine,<br>Newberry, Laura; Malawi-Liverpool-Wellcome Trust Clinical Research Programme, Department of Paediatrics and Child Health<br>Mambule, Ivan; Malawi-Liverpool-Wellcome Trust Clinical Research Programme<br>Everett, Dean; Malawi-Liverpool-Wellcome Trust Clinical Research Programme, Department of Paediatrics and Child Health; Khalifa University<br>Mwansambo, Charles; Government of Malawi Ministry of Health<br>Cunliffe, Nigel; University of Liverpool, Clinical Infection, Microbiology and Immunology<br>French, Neil; University of Liverpool Faculty of Health and Life Sciences, Centre for Global Vaccine Research, Institute of Infection and Global Health<br>Heyderman, Robert; University College London, Division of Infection and Immunity<br>Bar-Zeev, Naor ; Johns Hopkins University Bloomberg School of Public Health, Global Disease Epidemiology and Contro
**Primary Subject Heading**:	Paediatrics
Secondary Subject Heading:	Global health, HIV/AIDS, Respiratory medicine
Keywords:	Epidemiology < INFECTIOUS DISEASES, Paediatric infectious disease & immunisation < PAEDIATRICS, Respiratory infections < THORACIC MEDICINE

SCHOLARONE™
Manuscripts

I, the Submitting Author has the right to grant and does grant on behalf of all authors of the Work (as defined in the below author licence), an exclusive licence and/or a non-exclusive licence for contributions from authors who are: i) UK Crown employees; ii) where BMJ has agreed a CC-BY licence shall apply, and/or iii) in accordance with the terms applicable for US Federal Government officers or employees acting as part of their official duties; on a worldwide, perpetual, irrevocable, royalty-free basis to BMJ Publishing Group Ltd ("BMJ") its licensees and where the relevant Journal is co-owned by BMJ to the co-owners of the Journal, to publish the Work in this journal and any other BMJ products and to exploit all rights, as set out in our licence.

The Submitting Author accepts and understands that any supply made under these terms is made by BMJ to the Submitting Author unless you are acting as an employee on behalf of your employer or a postgraduate student of an affiliated institution which is paying any applicable article publishing charge ("APC") for Open Access articles. Where the Submitting Author wishes to make the Work available on an Open Access basis (and intends to pay the relevant APC), the terms of reuse of such Open Access shall be governed by a Creative Commons licence – details of these licences and which Creative Commons licence will apply to this Work are set out in our licence referred to above.

Other than as permitted in any relevant BMJ Author's Self Archiving Policies, I confirm this Work has not been accepted for publication elsewhere, is not being considered for publication elsewhere and does not duplicate material already published. I confirm all authors consent to publication of this Work and authorise the granting of this licence.

For peer review only

Clinical pneumonia in the hospitalised child in Malawi in the post-pneumococcal conjugate vaccine era: a prospective hospital-based observational study

Pui-Ying Iroh Tam,[1,2,3,a*] James Chirombo,[4*] Marc Y. R. Henrion,[3,4] Laura Newberry,[1,2,b] Ivan Mambule,[1,5,c] Dean Everett,[1,5,d] Charles Mwansambo,[6] Nigel A. Cunliffe,[5] Neil French,[5] Robert S. Heyderman,[7] and Naor Bar-Zeev[1,2,5,e] for the VacSurv Consortium

[1]Paediatrics and Child Health Research Group, Malawi-Liverpool-Wellcome Trust Clinical Research Programme, Blantyre, Malawi

[2]Department of Paediatrics and Child Health, University of Malawi College of Medicine, Blantyre, Malawi

[3]Department of Clinical Sciences, Liverpool School of Tropical Medicine, Liverpool, UK

[4]Statistical Support Unit, Malawi-Liverpool-Wellcome Trust Clinical Research Programme, Blantyre, Malawi

[5]Institute of Infection, Veterinary and Ecological Science, University of Liverpool, Liverpool, UK

[6]Malawi Ministry of Health, Lilongwe, Malawi

[7]Research Department of Infection, Division of Infection & Immunity, University College London, London, UK

*These authors contributed equally to the manuscript.

[a]Corresponding author: irohtam@mlw.mw

For peer review only

For peer review only - http://bmjopen.bmj.com/site/about/guidelines.xhtml

[b]Current address: Kaiser Permanente, Parker, CO, USA

[c]Current address: Medical Research Council, Kampala, Uganda

[d]Current address: Khalifa University, Abu Dhabi, UAE

[e]Current address: Johns Hopkins School of Public Health, Baltimore, MD, USA

Short title: Clinical pneumonia in the Malawian child

For peer review only

Abbreviations

CI, confidence interval

CPAP, continuous positive airway pressure

HEU, HIV-exposed, uninfected

HUU, HIV-unexposed, uninfected

IMCI, integrated management of childhood illness

IQR, interquartile range

mRISC, modified respiratory index of severity in children

MUAC, mid-upper arm circumference

PCV13, 13-valent pneumococcal conjugate vaccine

ResViNet, Respiratory Syncytial Virus Network

RISC, respiratory index of severity in children

For peer review only - http://bmjopen.bmj.com/site/about/guidelines.xhtml

Abstract

Objective

Assess characteristics of clinical pneumonia after introduction of pneumococcal conjugate vaccine (PCV), by HIV exposure status, in children hospitalized in a governmental hospital in Malawi.

Methods and findings

We evaluated 1,139 children ≤5 years old hospitalised with clinical pneumonia: 101 HIV-exposed uninfected (HEU) and 1038 HIV-unexposed, uninfected (HUU). Median age was 11 months (IQR 6-20), 59% were male, median mid-upper arm circumference (MUAC) was 14 cm (IQR 13-15) and mean weight-for-height z score was -0.7 (±2.5). The highest Respiratory Index of Severity in Children (RISC) scores were allocated to 10.4% of the overall cohort. Only 45.7% had fever, and 37.2% had at least one danger sign at presentation. The most common clinical features were crackles (54.7%), nasal flaring (53.5%), and lower chest wall indrawing (53.2%). Compared to HUU, HEU children were significantly younger (9 months v. 11 months), with lower mean birth weight (2.8 kg v. 3.0 kg) and MUAC (13.6 cm v. 14.0 cm), had higher prevalence of vomiting (32.7% v. 22.0%), tachypnoea (68.4% v. 49.8%), and highest RISC scores (20.0% v. 9.4%). Five children died (0.4%). However, clinical outcomes were similar for both groups.

Conclusions

For peer review only

In this post-PCV setting where prevalence of HIV and malnutrition is high, children hospitalised fulfilling the WHO Integrated Management of Childhood Illness criteria for clinical pneumonia present with heterogeneous features. These vary by HIV exposure status but this does not influence either the frequency of danger signs or mortality. The poor performance of available severity scores in this population and the absence of more specific diagnostics hinder appropriate antimicrobial stewardship and the rational application of other interventions.

266 words (max 250)

Keywords: children, HIV-exposed, pneumonia, predictive value, respiratory tract, risk score

For peer review only

Strengths and limitations of the study

- We evaluated over 1,100 children hospitalized with pneumonia in a low-income country setting after introduction of PCV.
- This observational cohort was nested within a prospective hospital-based study of PCV13 effectiveness
- We assessed the demographic and clinical characteristics of clinical pneumonia patients and compared HEU vs. HUU children, and computed RISC scores for severe pneumonia

For peer review only

For peer review only - http://bmjopen.bmj.com/site/about/guidelines.xhtml

Introduction

Pneumonia is the leading cause of morbidity and mortality in children globally. This disease is of particular importance in low-income countries such as Malawi, where HIV prevalence among pregnant women is 10.8% [1], and where a high burden of malaria and malnutrition (stunting prevalence of 39% [2]) contributes to an under-5 mortality rate of 55 per 1,000 live births [3].

WHO Integrated Management of Childhood Illness (IMCI) guidelines for community and referral level management of children with pneumonia rely upon clinical recognition of features of severe disease. WHO lists general danger signs, such as inability to drink, persistent vomiting, convulsions, lethargy or unconsciousness, or stridor in a calm child, which are clinical features used to identify children who should be treated in an inpatient setting and to receive injectable antibiotics [4]. However, clinical features are non-specific and can reflect a variety of other infectious (e.g. bronchiolitis, malaria, sepsis, tuberculosis, pneumocystis), and non-infectious (e.g. asthma, lymphocytic interstitial pneumonitis, pulmonary Kaposi sarcoma) conditions. Inappropriate and overuse of antimicrobials in low resource settings where antimicrobial prescribing is based more on clinical features than objective diagnostic data has implications for antimicrobial stewardship in the post-pneumococcal conjugate vaccine (PCV) era, particularly in settings where antimicrobial choice and supply is limited and antimicrobial resistance rates are rising [5].

Few risk scoring systems to predict severe pneumonia exist that are adaptable to low-income countries with high HIV prevalence – Respiratory Index of Severity in Children-Malawi (RISC-

For peer review only

Malawi) is the only score from children from a low-income country, and did not consider HIV status. Furthermore, although the alternative, Respiratory Index of Severity in Children (RISC) score does include a scoring system based on HIV infection, no studies have specifically evaluated whether HIV exposure status affects the presence and predictive value of clinical features for severe disease and case-fatality. As HIV-exposed, uninfected (HEU) children constitute a growing population and, compared to HIV-unexposed, uninfected (HUU) remain vulnerable to more severe pneumonia, more often fail empiric pneumonia treatment, and have higher rates of hospitalisation and death than HIV-unexposed, uninfected (HUU) children [6-9], this population is of growing clinical relevance. Our objectives were to describe the clinical features of children hospitalised with pneumonia, calculate risk scores for our population, and to determine whether differences in clinical characteristics and calculated risk scores exist by HIV exposure status.

Methods

This observational cohort was nested within a prospective hospital-based study of 13-valent PCV effectiveness conducted at Queen Elizabeth Central Hospital between 2 April 2013 and 3 August 2016. Children born on or after 1 October 2012 (based on verbal and written documentation from parents on time since birth) and therefore eligible for PCV and admitted with a diagnosis of clinical pneumonia were enrolled (Supplementary Table 1). Children were excluded if they had known oncological or congenital heart disease, were admitted ≥48 hours before recruitment, were re-admitted to hospital within 14 days of a previous hospitalisation, or presented moribund with impending death. Study staff performed active case finding Monday-Friday during daytime

For peer review only

in the emergency department and inpatient wards, and followed up study participants 6 weeks following hospital discharge with a home visit or phone call to confirm vital status.

With informed written parental consent, all enrolled children had baseline characteristics measured which included clinical, laboratory and sociodemographic variables. Oxygen saturation was measured with an appropriately sized sensor and a Nellcor pulse oximeter (Welch Allyn Spot Vital Signs Devices, Skaneateles Falls, NY, USA) in room air. Children with oxygen saturations <90% in room air were given supplemental oxygen via nasal cannula using an oxygen concentrator. At clinician decision and if a free circuit was available, children with severe respiratory distress, signs of exhaustion or worsening hypoxemia received bubble continuous positive airway pressure (Pumani, 3rd Stone Design, San Rafael, CA, USA). Blood was routinely collected for packed cell volume and malaria parasite thick film. Mothers and infants were tested for HIV according to the Malawi National HIV Guidelines [10]. A blood culture was obtained only when the admitting clinician suspected sepsis. Children with suspected meningitis, malaria or anaemia, were managed according to respective WHO and hospital treatment guidelines [11].

This study was approved by the University of Malawi College of Medicine Research Ethics Committee (P.05/13/1386) and by the University of Liverpool (RETH490).

Patient and public involvement

Patients or the public were not involved in the design, or conduct, or reporting, or dissemination plans of our research.

For peer review only - http://bmjopen.bmj.com/site/about/guidelines.xhtml

Case definition

Pneumonia was diagnosed clinically per WHO IMCI criteria.

Statistical analysis

We assessed the demographic and clinical characteristics of clinical pneumonia patients and compared HEU vs. HUU children using Fisher's exact test, two-sample t-test or Wilcoxon rank-sum tests respectively for categorical, continuous normal and continuous skewed variables.

We computed RISC scores for severe pneumonia in our cohort [12-14], using the variables listed in Table 1. We had insufficient variables to compute modified Respiratory Index of Severity in Children (mRISC) and RISC-Malawi scores [13].

Results

A total of 1660 children were recruited into the study, among whose files were incomplete for 521. We included 1,139 children in the analysis, of whom 101 were HEU and 1,038 HUU. The median age was 11 months (IQR 6.0, 20.0), 59% were male, and the median mid-upper arm circumference (MUAC) was 14 cm (IQR 13-15) and mean weight-for-height z score -0.7 (±2.5; Table 2 and Supplementary Table 2).

In our cohort, no single clinical feature was present at enrollment in more than 55% of the population: the most common clinical features were crackles (54.7%), nasal flaring (53.5%), and lower chest wall indrawing (53.2%). Less than half had fever (45.7%), and at least one IMCI

For peer review only - http://bmjopen.bmj.com/site/about/guidelines.xhtml

danger sign (37.2%). The median oxygen saturation at presentation was 95% (IQR 89,98), with 25.4% having oxygen saturations below 90%. Among this cohort, 33.9% received oxygen and 2.1% were placed on CPAP. The highest RISC scores (3+) were allocated to 10.4% of the overall cohort, respectively. Five children died (0.4%).

Ten percent of children in our cohort were HEU, and notable differences between HEU and HUU were observed in several areas. In the HEU group, children were younger (median age 9 months (IQR 5.0-15.5) v. 11 months (IQR 6.0-20.0), p=0.02), with lower birth weight (median 2.8 kg (IQR 1.8-3.2) v. 3.0 kg (IQR 2.4-3.3), p=0.03), lower weight-for-height z score (mean -1.2±2.5 vs. -0.6±2.5, p<0.01) and lower MUAC (median 13.6 cm (IQR 12.6-14.6) v. 14 cm (IQR 13-15), p<0.01). Compared to the HUU group, HEU children had significantly higher prevalence of vomiting (32.7% v. 22.0%, p=0.02) and age-defined tachypnoea in infancy (68.4% v. 49.8%, p=0.02). More HEU children presented with at least one IMCI danger sign (46.5% v. 36.3%, p=0.05). We did not observe significant differences in respiratory symptoms and in proportions of children who died between groups, although HEU children received oxygen at a significantly higher rate than HUU children (44.0% v. 32.9%, p=0.04). Clinical outcomes between the HEU and HUU groups were also similar (Table 2). However, RISC scores were significantly different between HIV exposure status, with 20.0% of HEU with the highest scores, compared to 9.4% of HUU (Fig 1). The risk score had a nonparametric distribution and many subjects scored 0.

Discussion

In our prospective cohort of children presenting with WHO IMCI criteria for clinical pneumonia in the post-PCV era, the most common clinical features were crackles, nasal flaring, and lower

For peer review only

chest wall indrawing. Despite the clinical diagnosis of pneumonia, features were heterogeneous. Thirty-four percent had an oxygen requirement and 2.1% needed CPAP support, though with best available treatment, overall mortality was low – and possibly a reflection of improvements in child health overall as a result of antiretroviral and antimalarial therapies, vaccination, and community management of nutrition. Ten percent of children hospitalised with pneumonia were HEU and this group was significantly younger, with lower birth weight and anthropometric parameters. HEU children had a significantly higher prevalence of vomiting, age-defined tachypnoea in infancy, and higher RISC scores, compared to HUU children.

Though HEU children were significantly younger and had significantly lower MUAC than HUU children, and therefore age and anthropometry are potential triage indicators for use in the clinical management of pneumonia, these differences were subtle and may not be useful for a clinician, who in low resource settings make decisions primarily on clinical suspicion. WHO IMCI clinical criteria for pneumonia are non-specific, and based on presence of cough, fast and difficult breathing. The non-specific nature of the diagnosis, which covers both infectious and non-infectious causes, is deliberately intended to increase sensitivity; however, as reflected in this study, this may have resulted in the term being used with a variety of features. Much research has been expended on developing pneumonia risk scores to identify children at risk of severe disease. However, the limitations of existing pneumonia risk scores for children in low- and middle-income country settings [15], including Pneumonia Etiology Research in Child Health (PERCH) [16], Respiratory Syncytial Virus Network (ReSViNet) [17], RISC [14], mRISC [12], and RISC-Malawi [13], is that the predictive capacity is generally modest and, in the case of PERCH, was found to be not as discriminatory as the WHO danger signs. The risk

For peer review only

scores we calculated in our cohort were not normally distributed and yielded surprisingly divergent results, indicating that they are limited to their study populations and are not generalisable.

In our cohort, the lack of predominating features of clinical pneumonia points to another possibility that, in post-PCV settings where HIV and chronic malnutrition rates are high (10% and 39%, respectively), children do not present with typical signs and symptoms. The difference in distribution of risk scores within our cohort highlights the fact that risk scores are population- and cohort-specific. In our cohort a substantial proportion of children met criteria for severe acute malnutrition (9.7% had weight-for-height z scores ≤3; and 4.1% had MUAC <11.5 cm), indicating how widespread chronic malnutrition is in Malawi. This pervasive underlying comorbidity leads to subsequent issues with identification and management of disease; and may in part explain overuse of antimicrobials in this setting, one factor contributing to an increase in antimicrobial resistance.

Antimicrobial stewardship and infection control issues in low-resource settings have been particularly problematic among young children, with significant increases in antimicrobial resistance noted among young infants with bloodstream infections over a twenty-year period [5], and concerns over resistance rates in childhood pneumonia in sub-Saharan Africa [18]. Challenges with diagnostic capacity and the limitations of clinical diagnosis hinder appropriate antimicrobial stewardship [19]. Risk scores that are adequately discriminatory therefore present an opportunity for targeted interventions, including early aggressive support, as well as judicious allocation of resources.

For peer review only - http://bmjopen.bmj.com/site/about/guidelines.xhtml

This study has several limitations. Only a very small proportion of HIV-exposed infants had HIV status tested by PCR, as recommended by national guidelines. This was due to shortage of HIV counselors, lack of testing reagents and reluctance of parents to obtain additional testing on their children. We did not recruit moribund children who died very soon after arrival, and therefore we may have underestimated our fatality rate. Demographics may play into admissions at a government tertiary hospital, and therefore there may be selection bias. A substantial proportion of recruited participants had incomplete files and were therefore not included in the analysis. Due to small numbers, we were unable to calculate predictive values for severe outcomes. However, this was a detailed prospective study conducted in a low resource setting in the post-PCV era where robust diagnostic testing was available.

In conclusion, in this setting where prevalence of HIV and malnutrition is high, children hospitalised fulfilling the WHO IMCI criteria for clinical pneumonia in the post-PCV era present with heterogeneous features. These vary by HIV exposure status but this does not influence either the frequency of danger signs or mortality. The sample size limited our ability to calculate predictive scores; and the performance of available severity scores in this population and the absence of more specific diagnostics hinder appropriate antimicrobial stewardship and the rational application of other interventions. Further work in developing antimicrobial stewardship scores in resource-limited settings, in a period defined by rising rates of antimicrobial resistance, may be warranted.

1,990 words

For peer review only - http://bmjopen.bmj.com/site/about/guidelines.xhtml

Acknowledgements

The authors wish to thank the collaborating members of the VacSurv Consortium (James Beard, Amelia C Crampin, Carina King, Sonia Lewycka, Hazzie Mvula, Tambosi Phiri, Miren Iturriza-Gomara, Osamu Nakagomi, Jennifer Verani, Cynthia Whitney).

Funding statement

This work was supported by a Wellcome Trust Programme Grant (Grant number 091909/Z/10/Z) and the MLW Programme Core Award (Grant 206454) from the Wellcome Trust. The funders had no role in study design, data collection and analysis, decision to publish, or preparation of the manuscript. The findings and conclusions in this report are those of the authors and do not necessarily represent the official position of the Centers for Disease Control and Prevention, Atlanta USA.

Competing interests

PI has received investigator-initiated research grant support from Bill & Melinda Gates Foundation outside the scope of this work; NAC has received research grant support and honoraria for participation in rotavirus vaccine advisory board meetings from GlaxoSmithKline Biologicals and from Takeda Pharmaceuticals outside the scope of this work; NF has received investigator-initiated research grant support from GlaxoSmithKline Biologicals and from Takeda Pharmaceuticals outside the scope of this work. NBZ has received investigator-initiated research grant support from GlaxoSmithKline Biologicals, Takeda Pharmaceuticals, Merck-Sharpe-

For peer review only

For peer review only - http://bmjopen.bmj.com/site/about/guidelines.xhtml

Dohme and the Serum Institute of India, all outside the scope of this work. All other authors declare that they have no financial disclosures or competing interests.

Author contributorship statement

Conceived and designed the research question: PI, NBZ; Conducted the statistical analysis: JC, MH; Managed the study: LN, IM; Developed and awarded funding for the study: DE, NAC, CM, NF, RH; Wrote the first draft of the manuscript: PI; Provided critical feedback on the manuscript: JC, MH, NAC, NF, RH, NBZ; Reviewed and approved the final draft: all authors.

Data availability

Data is available upon reasonable request to the corresponding author.

Ethics statement

This study was approved by the University of Malawi College of Medicine Research Ethics Committee (P.05/13/1386) and by the University of Liverpool (RETH490).

For peer review only

References

1. UNAIDS: **Malawi factsheet**. 2019.

2. World Bank: **Prevalence of stunting - Malawi**. 2018.

3. WHO: **Malawi Maternal and Child Health Data**. Geneva, Switzerland: WHO; 2015.

4. WHO: **Revised WHO classification and treatment of childhood pneumonia at health facilities**. Geneva, Switzerland: WHO; 2014.

5. Iroh Tam PY, Musicha P, Kawaza K, Cornick J, Denis B, Freyne B, Everett D, Dube Q, French N, Feasey N *et al*: **Emerging Resistance to Empiric Antimicrobial Regimens for Pediatric Bloodstream Infections in Malawi (1998-2017)**. *Clin Infect Dis* 2019, **69**(1):61-68.

6. Landes M, van Lettow M, Chan AK, Mayuni I, Schouten EJ, Bedell RA: **Mortality and health outcomes of HIV-exposed and unexposed children in a PMTCT cohort in Malawi**. *PLoS One* 2012, **7**(10):e47337.

7. Slogrove A, Reikie B, Naidoo S, De Beer C, Ho K, Cotton M, Bettinger J, Speert D, Esser M, Kollmann T: **HIV-exposed uninfected infants are at increased risk for severe infections in the first year of life**. *J Trop Pediatr* 2012, **58**(6):505-508.

8. Slogrove AL, Goetghebuer T, Cotton MF, Singer J, Bettinger JA: **Pattern of Infectious Morbidity in HIV-Exposed Uninfected Infants and Children**. *Front Immunol* 2016, **7**:164.

For peer review only - http://bmjopen.bmj.com/site/about/guidelines.xhtml

9. Zar HJ, Barnett W, Stadler A, Gardner-Lubbe S, Myer L, Nicol MP: **Aetiology of childhood pneumonia in a well vaccinated South African birth cohort: a nested case-control study of the Drakenstein Child Health Study**. *Lancet Respir Med* 2016, **4**(6):463-472.

10. Malawi Ministry of Health: **Clinical Management of HIV in Adults and Children**. Malawi; 2011.

11. WHO: **Handbook IMCI. Integrated management of childhood illness**. In. Geneva, Switzerland: World Health Organization; 2000.

12. Emukule GO, McMorrow M, Ulloa C, Khagayi S, Njuguna HN, Burton D, Montgomery JM, Muthoka P, Katz MA, Breiman RF *et al*: **Predicting mortality among hospitalized children with respiratory illness in Western Kenya, 2009-2012**. *PLoS One* 2014, **9**(3):e92968.

13. Hooli S, Colbourn T, Lufesi N, Costello A, Nambiar B, Thammasitboon S, Makwenda C, Mwansambo C, McCollum ED, King C: **Predicting Hospitalised Paediatric Pneumonia Mortality Risk: An External Validation of RISC and mRISC, and Local Tool Development (RISC-Malawi) from Malawi**. *PLoS One* 2016, **11**(12):e0168126.

14. Reed C, Madhi SA, Klugman KP, Kuwanda L, Ortiz JR, Finelli L, Fry AM: **Development of the Respiratory Index of Severity in Children (RISC) score among young children with respiratory infections in South Africa**. *PLoS One* 2012, **7**(1):e27793.

15. Deardorff KV, McCollum ED, Ginsburg AS: **Pneumonia Risk Stratification Scores for Children in Low-Resource Settings: A Systematic Literature Review**. *Pediatr Infect Dis J* 2018, **37**(8):743-748.

For peer review only

For peer review only - http://bmjopen.bmj.com/site/about/guidelines.xhtml

16. Gallagher KE, Knoll MD, Prosperi C, Baggett HC, Brooks WA, Feikin DR, Hammitt LL, Howie SRC, Kotloff KL, Levine OS *et al*: **The Predictive Performance of a Pneumonia Severity Score in Human Immunodeficiency Virus-negative Children Presenting to Hospital in 7 Low- and Middle-income Countries**. *Clin Infect Dis* 2020, **70**(6):1050-1057.

17. Justicia-Grande AJ, Pardo-Seco J, Cebey-Lopez M, Vilanova-Trillo L, Gomez-Carballa A, Rivero-Calle I, Puente-Puig M, Curros-Novo C, Gomez-Rial J, Salas A *et al*: **Development and Validation of a New Clinical Scale for Infants with Acute Respiratory Infection: The ReSVinet Scale**. *PLoS One* 2016, **11**(6):e0157665.

18. Iroh Tam PY, Sadoh AE, Obaro SK: **A meta-analysis of antimicrobial susceptibility profiles for pneumococcal pneumonia in sub-Saharan Africa**. *Paediatr Int Child Health* 2018, **38**(1):7-15.

19. Iroh Tam PY: **The Challenge and Opportunity of Pediatric Antimicrobial Stewardship in Low Resource Settings**. *J Trop Pediatr* 2020, **66**(1):1-3.

For peer review only

Table 1. List of variables used to compute RISC scores

RISC	Score
Oxygen saturation ≤90%	3
Chest indrawing	2
Refusal to feed	1
Wheezing	-2
WHO weight for age z-score ≤ -3	2
WHO weight for age z-score -3 ≤_z < -2	1

RISC, Respiratory Index of Severity in Children

For peer review only

Table 2. Characteristics of children hospitalised with clinical pneumonia, stratified by HIV exposure status

Characteristics	All (N=1139)	Group: HEU (N=101)	Group: HUU (N=1038)	p-value
**Demographic and physiological characteristics**				
Age, months (m)				0.02†
N	1118	99	1019	
Median (IQR)	11.0 (6.0, 20.0)	9.0 (5.0, 15.5)	11.0 (6.0, 20.0)	
Sex				0.92*
N	1139	101	1038	
Male (%)	677 (59.4)	61 (60.4)	616 (59.3)	
Birth weight, kg				0.03†
N	1117	97	1020	
Median (IQR)	3.0 (2.3, 3.3)	2.8 (1.8, 3.2)	3.0 (2.4, 3.3)	
Weight for height				< 0.01¥
N	1098	100	998	
Mean (SD)	-0.7 (2.5)	-1.2 (2.5)	-0.6 (2.5)	
MUAC, cm				< 0.01†
N	1129	101	1028	
Median (IQR)	14.0 (13.0, 15.0)	13.6 (12.6, 14.6)	14.0 (13.0, 15.0)	
Temperature				0.02†
N	1133	100	1033	
Median (IQR)	37.8 (36.9, 38.6)	38.0 (37.2, 39.0)	37.8 (36.8, 38.5)	
Fever				0.06
N	1133	100	1033	
Yes (%)	518 (45.7)	55 (55.0)	463 (44.8)	
Antibiotic use				0.34*
N	1131	101	1030	
Yes (%)	451 (39.9)	45 (44.6)	406 (39.4)	
Inability to drink				0.08*
N	1134	101	1033	
Yes (%)	91 (8.0)	13 (12.9)	78 (7.6)	
Vomiting				0.02*
N	1137	101	1036	
Yes (%)	261 (23.0)	33 (32.7)	228 (22.0)	
At least one IMCI danger sign				0.05*
N	1139	101	1038	
Yes (%)	424 (37.2)	47 (46.5)	377 (36.3)	

For peer review only

**Clinical features**				
Nasal flaring				0.92*
N	1137	101	1036	
Yes (%)	608 (53.5)	55 (54.5)	553 (53.4)	
Lower chest wall indrawing				1.00*
N	1137	101	1036	
Yes (%)	605 (53.2)	54 (53.5)	551 (53.2)	
Stridor				0.44*
N	1137	101	1036	
Yes (%)	22 (1.9)	3 (3.0)	19 (1.8)	
Age-defined tachypnoea				0.02*
N	666	57	609	
Age 0-12m and >50 breaths/min	342 (51.4)	39 (68.4)	303 (49.8)	
Age 12-60m and >40 breaths/min	324 (48.7)	18 (31.6)	306 (50.3)	
Oral thrush/sores				0.11*
N	1138	101	1037	
Yes (%)	21 (1.9)	4 (4.0)	17 (1.6)	
Hypoxemia <90%				0.40*
N	1128	100	1028	
Yes (%)	286 (25.4)	29 (29.0)	257 (25.0)	
**Risk scores**				
RISC				<0.01*
N	1120	100	1020	
-2 to 2	1004 (89.6)	80 (80.0)	924 (90.6)	
3-6	116 (10.4)	20 (20.0)	96 (9.4)	
**Clinical management**				
Received oxygen				0.04*
N	1129	100	1029	
Yes (%)	383 (33.9)	44 (44.0)	339 (32.9)	
Placed on CPAP				0.14*
N	1118	99	1019	
Yes (%)	23 (2.1)	4 (4.0)	19 (1.9)	
**Outcomes**				
Length of hospital stay, days				0.21‡
N	1086	96	990	
Median (IQR)	2.0 (2.0, 4.0)	2.0 (2.0, 4.0)	2.0 (2.0, 3.0)	
Mortality				0.07*
N	1139	101	1038	
Yes (%)	5 (0.4)	2 (2.0)	3 (0.3)	

For peer review only

CPAP, continuous positive airway pressure; HEU, HIV-exposed, uninfected; HUU, HIV-unexposed, uninfected; IMCI, Integrated Management of Childhood Illness; IQR, interquartile range; MUAC, mid-upper arm circumference; RISC, Respiratory Index of Severity in Children

* *Fisher's exact test*

† Wilcoxon rank sum test

¥ Independent two sample t-test

For peer review only - http://bmjopen.bmj.com/site/about/guidelines.xhtml

Figure legend

Fig 1. Summary of RISC scores computed from the data

RISC, Respiratory Index of Severity in Children

Appendix

Supplementary Table 1. STROBE checklist

Supplementary Table 2. Full characteristics of children hospitalised with clinical pneumonia, stratified by HIV exposure status

For peer review only

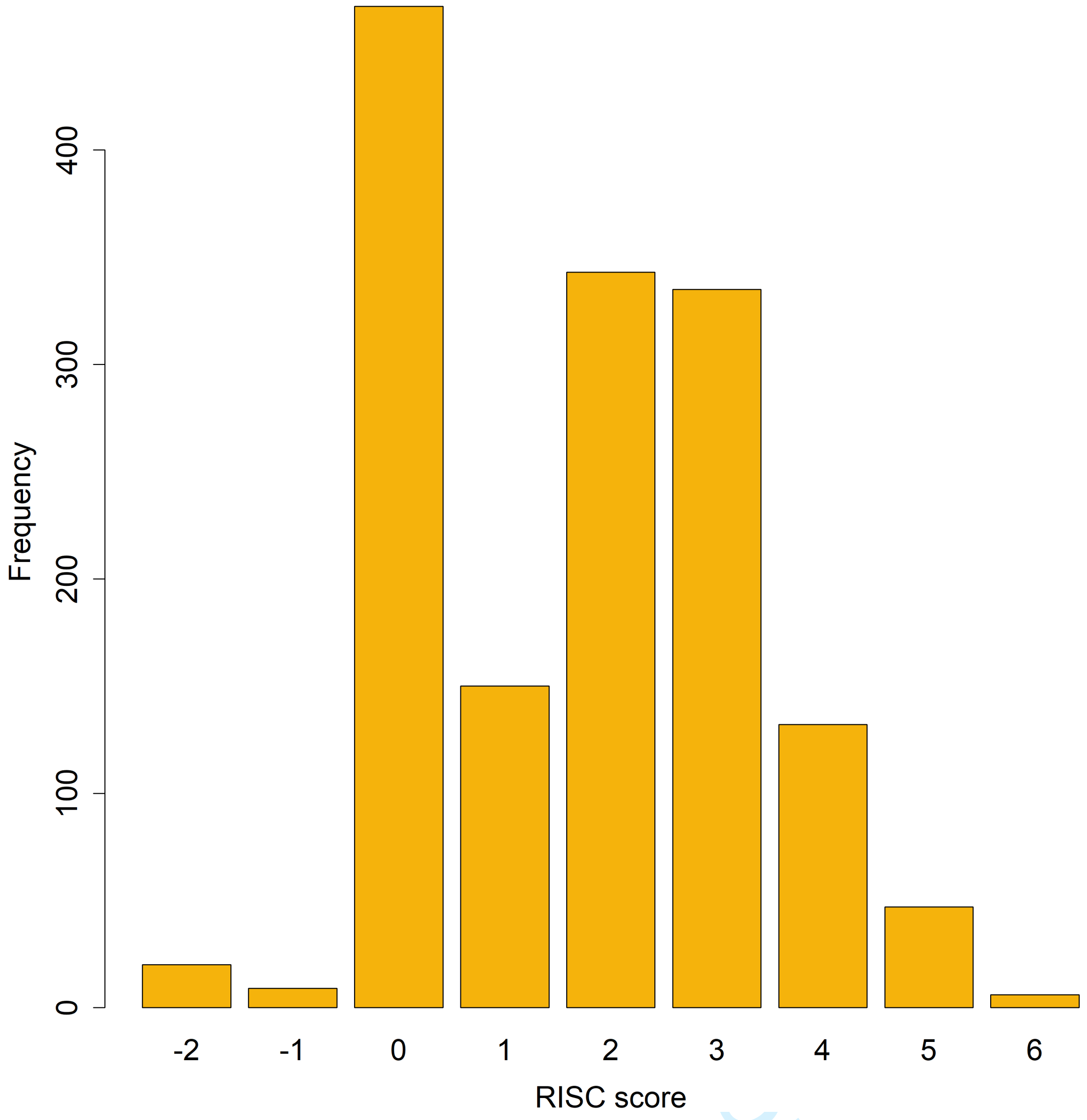
Frequency
0
100
200
300
400
-2
-1
0
1
2
3
4
5
6
RISC score

Supplementary Table 1. STROBE Statement—Checklist of items that should be included in reports of ***cohort studies***

	Item No	Recommendation	Page
**Title and abstract**	1	(*a*) Indicate the study's design with a commonly used term in the title or the abstract	1
		(*b*) Provide in the abstract an informative and balanced summary of what was done and what was found	3
**Introduction**			
Background/rationale	2	Explain the scientific background and rationale for the investigation being reported	6
Objectives	3	State specific objectives, including any prespecified hypotheses	7
**Methods**			
Study design	4	Present key elements of study design early in the paper	7
Setting	5	Describe the setting, locations, and relevant dates, including periods of recruitment, exposure, follow-up, and data collection	7
Participants	6	(*a*) Give the eligibility criteria, and the sources and methods of selection of participants. Describe methods of follow-up	7
		(*b*) For matched studies, give matching criteria and number of exposed and unexposed	N/A
Variables	7	Clearly define all outcomes, exposures, predictors, potential confounders, and effect modifiers. Give diagnostic criteria, if applicable	8
Data sources/ measurement	8*	For each variable of interest, give sources of data and details of methods of assessment (measurement). Describe comparability of assessment methods if there is more than one group	8
Bias	9	Describe any efforts to address potential sources of bias	9
Study size	10	Explain how the study size was arrived at	9
Quantitative variables	11	Explain how quantitative variables were handled in the analyses. If applicable, describe which groupings were chosen and why	9
Statistical methods	12	(*a*) Describe all statistical methods, including those used to control for confounding	9
		(*b*) Describe any methods used to examine subgroups and interactions	9
		(*c*) Explain how missing data were addressed	9
		(*d*) If applicable, explain how loss to follow-up was addressed	9
		(*e*) Describe any sensitivity analyses	N/A
**Results**			
Participants	13*	(a) Report numbers of individuals at each stage of study—eg numbers potentially eligible, examined for eligibility, confirmed eligible, included in the study, completing follow-up, and analysed	9
		(b) Give reasons for non-participation at each stage	9
		(c) Consider use of a flow diagram	N/A

For peer review only - http://bmjopen.bmj.com/site/about/guidelines.xhtml

Descriptive data	14*	(a) Give characteristics of study participants (eg demographic, clinical, social) and information on exposures and potential confounders	Table 2
		(b) Indicate number of participants with missing data for each variable of interest	9
		(c) Summarise follow-up time (eg, average and total amount)	9
Outcome data	15*	Report numbers of outcome events or summary measures over time	9
Main results	16	(*a*) Give unadjusted estimates and, if applicable, confounder-adjusted estimates and their precision (eg, 95% confidence interval). Make clear which confounders were adjusted for and why they were included	9-10
		(*b*) Report category boundaries when continuous variables were categorized	Table 2
		(*c*) If relevant, consider translating estimates of relative risk into absolute risk for a meaningful time period	N/A
Other analyses	17	Report other analyses done—eg analyses of subgroups and interactions, and sensitivity analyses	N/A
**Discussion**			
Key results	18	Summarise key results with reference to study objectives	10-11
Limitations	19	Discuss limitations of the study, taking into account sources of potential bias or imprecision. Discuss both direction and magnitude of any potential bias	13
Interpretation	20	Give a cautious overall interpretation of results considering objectives, limitations, multiplicity of analyses, results from similar studies, and other relevant evidence	13
Generalisability	21	Discuss the generalisability (external validity) of the study results	13
**Other information**			
Funding	22	Give the source of funding and the role of the funders for the present study and, if applicable, for the original study on which the present article is based	14

*Give information separately for exposed and unexposed groups.

Note: An Explanation and Elaboration article discusses each checklist item and gives methodological background and published examples of transparent reporting. The STROBE checklist is best used in conjunction with this article (freely available on the Web sites of PLoS Medicine at http://www.plosmedicine.org/, Annals of Internal Medicine at http://www.annals.org/, and Epidemiology at http://www.epidem.com/). Information on the STROBE Initiative is available at http://www.strobe-statement.org.

For peer review only

Supplementary Table 2. Full characteristics of children hospitalised with clinical pneumonia, stratified by HIV exposure status

Characteristics	All (N=1139)	Group		p-value
		HEU (N=101)	HUU (N=1038)	
**Demographic and physiological characteristics**				
Age group				
N	1116	99	1017	
0-2 m	126 (11.3)	13 (13.1)	113 (11.1)	
2-6 m	188 (16.9)	23 (23.2)	165 (16.2)	
6-12 m	302 (27.1)	32 (32.3)	270 (26.6)	
12-24 m	318 (28.5)	18 (18.2)	300 (29.5)	
24-60 m	182 (16.3)	13 (13.1)	169 (16.6)	
Low birth weight				0.04*
N	1117	97	1020	
< 2.5 kg (%)	302 (27.0)	35 (36.1)	267 (26.2)	
Weight for height categories				
N	1098	101	998	
<-3 (%)	106 (9.7)	15 (15. 0)	91 (9.1)	
-3- -2 (%)	248 (0.9)	31 (31.0)	217 (21.7)	
>-2 (%)	1102 (98.6)	54 (54.0)	690 (69.1)	
MUAC categories				
N	1139	101	1038	
< 11.5 cm (%)	47 (4.1)	13 (12.9)	34 (3.3)	
Previous visit to health centre				1.00*
N	1139	101	1038	
Yes (%)	979 (86.0)	87 (86.1)	892 (85.9)	
**Household characteristics**				
Household size >4				0.83*
N	1136	101	1035	
Yes (%)	480 (42.3)	44 (43.6)	436 (42.1)	
**Clinical features**				
Crackles				0.75*
N	1139	101	1038	
Yes (%)	623 (54.7)	57 (56.4)	566 (54.5)	
Wheezing				0.41*
N	1137	101	1036	
Yes (%)	192 (16.9)	20 (19.8)	172 (16.6)	
Bronchial breathing				0.12*
N	1139	101	1038	
Yes (%)	70 (6.2)	10 (9.9)	60 (5.8)	
Reduced air entry				0.19*
N	1139	101	1038	

For peer review only

Yes	9 (0.8)	2 (2.0)	7 (0.7)	
Age-adjusted tachycardia				0.11*
N	505	46	459	
Age 0-12m and >160 beats/min	266 (52.7)	31 (67.4)	235 (51.2)	
Age 12-36m and >150 beats/min	204 (40.4)	14 (30.4)	190 (41.4)	
Age 36-60m and >140 beats/min	35 (6.9)	1 (2.2)	34 (7.4)	
Hepatomegaly				0.61*
N	1135	99	1036	
Yes (%)	11 (1.0)	0 (0.0)	11 (1.1)	
Oxygen saturation				0.28†
N	1128	100	1028	
Median (IQR)	95.0 (89.0, 98.0)	95.0 (86.0, 98.0)	95.0 (89.8, 98.0)	
**Clinical management**				
Received IVF				1.00*
N	1120	100	1020	
Yes (%)	14 (1.3)	1 (1.0)	13 (1.3)	

HEU, HIV-exposed, uninfected; HUU, HIV-unexposed, uninfected; IQR, interquartile range;

IVF, intravenous fluids; MUAC, mid-upper arm circumference

* *Fisher's exact test*

† Wilcoxon rank sum test

For peer review only

BMJ Open

Clinical pneumonia in the hospitalised child in Malawi in the post-pneumococcal conjugate vaccine era: a prospective hospital-based observational study

Journal:	*BMJ Open*
Manuscript ID	bmjopen-2021-050188.R3
Article Type:	Original research
Date Submitted by the Author:	20-Nov-2021
Complete List of Authors:	Iroh Tam, Pui-Ying ; Malawi-Liverpool-Wellcome Trust Clinical Research Programme, Department of Paediatrics and Child Health; Liverpool School of Tropical Medicine, Department of Paediatrics and Child Health<br>Chirombo, James; Malawi-Liverpool-Wellcome Trust Clinical Research Programme<br>Henrion, Marc; Malawi-Liverpool-Wellcome Trust Clinical Research Programme; Liverpool School of Tropical Medicine,<br>Newberry, Laura; Malawi-Liverpool-Wellcome Trust Clinical Research Programme, Department of Paediatrics and Child Health<br>Mambule, Ivan; Malawi-Liverpool-Wellcome Trust Clinical Research Programme<br>Everett, Dean; Malawi-Liverpool-Wellcome Trust Clinical Research Programme, Department of Paediatrics and Child Health; Khalifa University<br>Mwansambo, Charles; Government of Malawi Ministry of Health<br>Cunliffe, Nigel; University of Liverpool, Clinical Infection, Microbiology and Immunology<br>French, Neil; University of Liverpool Faculty of Health and Life Sciences, Centre for Global Vaccine Research, Institute of Infection and Global Health<br>Heyderman, Robert; University College London, Division of Infection and Immunity<br>Bar-Zeev, Naor ; Johns Hopkins University Bloomberg School of Public Health, Global Disease Epidemiology and Contro
**Primary Subject Heading**:	Paediatrics
Secondary Subject Heading:	Global health, HIV/AIDS, Respiratory medicine
Keywords:	Epidemiology < INFECTIOUS DISEASES, Paediatric infectious disease & immunisation < PAEDIATRICS, Respiratory infections < THORACIC MEDICINE

For peer review only - http://bmjopen.bmj.com/site/about/guidelines.xhtml

I, the Submitting Author has the right to grant and does grant on behalf of all authors of the Work (as defined in the below author licence), an exclusive licence and/or a non-exclusive licence for contributions from authors who are: i) UK Crown employees; ii) where BMJ has agreed a CC-BY licence shall apply, and/or iii) in accordance with the terms applicable for US Federal Government officers or employees acting as part of their official duties; on a worldwide, perpetual, irrevocable, royalty-free basis to BMJ Publishing Group Ltd ("BMJ") its licensees and where the relevant Journal is co-owned by BMJ to the co-owners of the Journal, to publish the Work in this journal and any other BMJ products and to exploit all rights, as set out in our licence.

The Submitting Author accepts and understands that any supply made under these terms is made by BMJ to the Submitting Author unless you are acting as an employee on behalf of your employer or a postgraduate student of an affiliated institution which is paying any applicable article publishing charge ("APC") for Open Access articles. Where the Submitting Author wishes to make the Work available on an Open Access basis (and intends to pay the relevant APC), the terms of reuse of such Open Access shall be governed by a Creative Commons licence – details of these licences and which Creative Commons licence will apply to this Work are set out in our licence referred to above.

Other than as permitted in any relevant BMJ Author's Self Archiving Policies, I confirm this Work has not been accepted for publication elsewhere, is not being considered for publication elsewhere and does not duplicate material already published. I confirm all authors consent to publication of this Work and authorise the granting of this licence.

For peer review only

Clinical pneumonia in the hospitalised child in Malawi in the post-pneumococcal conjugate vaccine era: a prospective hospital-based observational study

Pui-Ying Iroh Tam,[1,2,3,a*] James Chirombo,[4*] Marc Y. R. Henrion,[3,4] Laura Newberry,[1,2,b] Ivan Mambule,[1,5,c] Dean Everett,[1,5,d] Charles Mwansambo,[6] Nigel A. Cunliffe,[5] Neil French,[5] Robert S. Heyderman,[7] and Naor Bar-Zeev[1,2,5,e] for the VacSurv Consortium

[1]Paediatrics and Child Health Research Group, Malawi-Liverpool-Wellcome Trust Clinical Research Programme, Blantyre, Malawi

[2]Department of Paediatrics and Child Health, University of Malawi College of Medicine, Blantyre, Malawi

[3]Department of Clinical Sciences, Liverpool School of Tropical Medicine, Liverpool, UK

[4]Statistical Support Unit, Malawi-Liverpool-Wellcome Trust Clinical Research Programme, Blantyre, Malawi

[5]Institute of Infection, Veterinary and Ecological Science, University of Liverpool, Liverpool, UK

[6]Malawi Ministry of Health, Lilongwe, Malawi

[7]Research Department of Infection, Division of Infection & Immunity, University College London, London, UK

*These authors contributed equally to the manuscript.

[a]Corresponding author: irohtam@mlw.mw

For peer review only

For peer review only - http://bmjopen.bmj.com/site/about/guidelines.xhtml

[b]Current address: Kaiser Permanente, Parker, CO, USA

[c]Current address: Medical Research Council, Kampala, Uganda

[d]Current address: Khalifa University, Abu Dhabi, UAE

[e]Current address: Johns Hopkins School of Public Health, Baltimore, MD, USA

Short title: Clinical pneumonia in the Malawian child

Abbreviations

CI, confidence interval

CPAP, continuous positive airway pressure

HEU, HIV-exposed, uninfected

HUU, HIV-unexposed, uninfected

IMCI, integrated management of childhood illness

IQR, interquartile range

mRISC, modified respiratory index of severity in children

MUAC, mid-upper arm circumference

PCV13, 13-valent pneumococcal conjugate vaccine

ResViNet, Respiratory Syncytial Virus Network

RISC, respiratory index of severity in children

For peer review only - http://bmjopen.bmj.com/site/about/guidelines.xhtml

Abstract

Objective

Assess characteristics of clinical pneumonia after introduction of pneumococcal conjugate vaccine (PCV), by HIV exposure status, in children hospitalized in a governmental hospital in Malawi.

Methods and findings

We evaluated 1,139 children ≤5 years old hospitalised with clinical pneumonia: 101 HIV-exposed uninfected (HEU) and 1038 HIV-unexposed, uninfected (HUU). Median age was 11 months (IQR 6-20), 59% were male, median mid-upper arm circumference (MUAC) was 14 cm (IQR 13-15) and mean weight-for-height z score was -0.7 (±2.5). The highest Respiratory Index of Severity in Children (RISC) scores were allocated to 10.4% of the overall cohort. Only 45.7% had fever, and 37.2% had at least one danger sign at presentation. The most common clinical features were crackles (54.7%), nasal flaring (53.5%), and lower chest wall indrawing (53.2%). Compared to HUU, HEU children were significantly younger (9 months v. 11 months), with lower mean birth weight (2.8 kg v. 3.0 kg) and MUAC (13.6 cm v. 14.0 cm), had higher prevalence of vomiting (32.7% v. 22.0%), tachypnoea (68.4% v. 49.8%), and highest RISC scores (20.0% v. 9.4%). Five children died (0.4%). However, clinical outcomes were similar for both groups.

Conclusions

In this post-PCV setting where prevalence of HIV and malnutrition is high, children hospitalised fulfilling the WHO Integrated Management of Childhood Illness criteria for clinical pneumonia

For peer review only

present with heterogeneous features. These vary by HIV exposure status but this does not influence either the frequency of danger signs or mortality. The poor performance of available severity scores in this population and the absence of more specific diagnostics hinder appropriate antimicrobial stewardship and the rational application of other interventions.

266 words (max 250)

Keywords: children, HIV-exposed, pneumonia, predictive value, respiratory tract, risk score

For peer review only

For peer review only - http://bmjopen.bmj.com/site/about/guidelines.xhtml

Strengths and limitations of the study

- We evaluated over 1,100 children hospitalized with pneumonia in a low-income country setting after introduction of PCV.
- This observational cohort was nested within a prospective hospital-based study of PCV13 effectiveness.
- We assessed the demographic and clinical characteristics of clinical pneumonia patients and compared HEU vs. HUU children, and computed RISC scores for severe pneumonia.
- This study was limited by the low mortality rate, small proportion of HIV-exposed infants with HIV status tested by PCR, the single-center study, a substantial proportion of incomplete files, and lack of recruitment of moribund children who died very soon after arrival into the study. However, this was a detailed prospective study conducted in a low resource setting in the post-PCV era where robust diagnostic testing was available.

For peer review only

For peer review only - http://bmjopen.bmj.com/site/about/guidelines.xhtml

Introduction

Pneumonia is the leading cause of morbidity and mortality in children globally. This disease is of particular importance in low-income countries such as Malawi, where HIV prevalence among pregnant women is 10.8% [1], and where a high burden of malaria and malnutrition (stunting prevalence of 39% [2]) contributes to an under-5 mortality rate of 55 per 1,000 live births [3].

WHO Integrated Management of Childhood Illness (IMCI) guidelines for community and referral level management of children with pneumonia rely upon clinical recognition of features of severe disease. WHO lists general danger signs, such as inability to drink, persistent vomiting, convulsions, lethargy or unconsciousness, or stridor in a calm child, which are clinical features used to identify children who should be treated in an inpatient setting and to receive injectable antibiotics [4]. However, clinical features are non-specific and can reflect a variety of other infectious (e.g. bronchiolitis, malaria, sepsis, tuberculosis, pneumocystis), and non-infectious (e.g. asthma, lymphocytic interstitial pneumonitis, pulmonary Kaposi sarcoma) conditions. Inappropriate and overuse of antimicrobials in low resource settings where antimicrobial prescribing is based more on clinical features than objective diagnostic data has implications for antimicrobial stewardship in the post-pneumococcal conjugate vaccine (PCV) era, particularly in

For peer review only - http://bmjopen.bmj.com/site/about/guidelines.xhtml

settings where antimicrobial choice and supply is limited and antimicrobial resistance rates are rising [5].

Few risk scoring systems to predict severe pneumonia exist that are adaptable to low-income countries with high HIV prevalence – Respiratory Index of Severity in Children-Malawi (RISC-Malawi) is the only score from children from a low-income country, and did not consider HIV status. Furthermore, although the alternative, Respiratory Index of Severity in Children (RISC) score does include a scoring system based on HIV infection, no studies have specifically evaluated whether HIV exposure status affects the presence and predictive value of clinical features for severe disease and case-fatality. As HIV-exposed, uninfected (HEU) children constitute a growing population and, compared to HIV-unexposed, uninfected (HUU) remain vulnerable to more severe pneumonia, more often fail empiric pneumonia treatment, and have higher rates of hospitalisation and death than HIV-unexposed, uninfected (HUU) children [6-9], this population is of growing clinical relevance. Our objectives were to describe the clinical features of children hospitalised with pneumonia, calculate risk scores for our population, and to determine whether differences in clinical characteristics and calculated risk scores exist by HIV exposure status.

Methods

This observational cohort was nested within a prospective hospital-based study of 13-valent PCV effectiveness conducted at Queen Elizabeth Central Hospital between 2 April 2013 and 3 August 2016. Children born on or after 1 October 2012 (based on verbal and written documentation from parents on time since birth) and therefore eligible for PCV and admitted with a diagnosis of

For peer review only

For peer review only - http://bmjopen.bmj.com/site/about/guidelines.xhtml

clinical pneumonia were enrolled (Supplementary Table 1). Children were excluded if they had known oncological or congenital heart disease, were admitted ≥48 hours before recruitment, were re-admitted to hospital within 14 days of a previous hospitalisation, or presented moribund with impending death. Study staff performed active case finding Monday-Friday during daytime in the emergency department and inpatient wards, and followed up study participants 6 weeks following hospital discharge with a home visit or phone call to confirm vital status.

With informed written parental consent, all enrolled children had baseline characteristics measured which included clinical, laboratory and sociodemographic variables. Oxygen saturation was measured with an appropriately sized sensor and a Nellcor pulse oximeter (Welch Allyn Spot Vital Signs Devices, Skaneateles Falls, NY, USA) in room air. Children with oxygen saturations <90% in room air were given supplemental oxygen via nasal cannula using an oxygen concentrator. At clinician decision and if a free circuit was available, children with severe respiratory distress, signs of exhaustion or worsening hypoxemia received bubble continuous positive airway pressure (Pumani, 3rd Stone Design, San Rafael, CA, USA). Blood was routinely collected for packed cell volume and malaria parasite thick film. Mothers and infants were tested for HIV according to the Malawi National HIV Guidelines [10]. A blood culture was obtained only when the admitting clinician suspected sepsis. Children with suspected meningitis, malaria or anaemia, were managed according to respective WHO and hospital treatment guidelines [11].

This study was approved by the University of Malawi College of Medicine Research Ethics Committee (P.05/13/1386) and by the University of Liverpool (RETH490).

For peer review only

For peer review only - http://bmjopen.bmj.com/site/about/guidelines.xhtml

Patient and public involvement

Patients or the public were not involved in the design, or conduct, or reporting, or dissemination plans of our research.

Case definition

Pneumonia was diagnosed clinically per WHO IMCI criteria.

Statistical analysis

We assessed the demographic and clinical characteristics of clinical pneumonia patients and compared HEU vs. HUU children using Fisher's exact test, two-sample t-test or Wilcoxon rank-sum tests respectively for categorical, continuous normal and continuous skewed variables.

We computed RISC scores for severe pneumonia in our cohort [12-14], using the variables listed in Table 1. We had insufficient variables to compute modified Respiratory Index of Severity in Children (mRISC) and RISC-Malawi scores [13].

Results

A total of 1660 children were recruited into the study, among whose files were incomplete for 521. We included 1,139 children in the analysis, of whom 101 were HEU and 1,038 HUU. The median age was 11 months (IQR 6.0, 20.0), 59% were male, and the median mid-upper arm circumference (MUAC) was 14 cm (IQR 13-15) and mean weight-for-height z score -0.7 (±2.5; Table 2 and Supplementary Table 2).

For peer review only - http://bmjopen.bmj.com/site/about/guidelines.xhtml

In our cohort, no single clinical feature was present at enrollment in more than 55% of the population: the most common clinical features were crackles (54.7%), nasal flaring (53.5%), and lower chest wall indrawing (53.2%). Less than half had fever (45.7%), and at least one IMCI danger sign (37.2%). The median oxygen saturation at presentation was 95% (IQR 89,98), with 25.4% having oxygen saturations below 90%. Among this cohort, 33.9% received oxygen and 2.1% were placed on CPAP. The highest RISC scores (3+) were allocated to 10.4% of the overall cohort, respectively. Five children died (0.4%).

Ten percent of children in our cohort were HEU, and notable differences between HEU and HUU were observed in several areas. In the HEU group, children were younger (median age 9 months (IQR 5.0-15.5) v. 11 months (IQR 6.0-20.0), p=0.02), with lower birth weight (median 2.8 kg (IQR 1.8-3.2) v. 3.0 kg (IQR 2.4-3.3), p=0.03), lower weight-for-height z score (mean -1.2±2.5 vs. -0.6±2.5, p<0.01) and lower MUAC (median 13.6 cm (IQR 12.6-14.6) v. 14 cm (IQR 13-15), p<0.01). Compared to the HUU group, HEU children had significantly higher prevalence of vomiting (32.7% v. 22.0%, p=0.02) and age-defined tachypnoea in infancy (68.4% v. 49.8%, p=0.02). More HEU children presented with at least one IMCI danger sign (46.5% v. 36.3%, p=0.05). We did not observe significant differences in respiratory symptoms and in proportions of children who died between groups, although HEU children received oxygen at a significantly higher rate than HUU children (44.0% v. 32.9%, p=0.04). Clinical outcomes between the HEU and HUU groups were also similar (Table 2). However, RISC scores were significantly different between HIV exposure status, with 20.0% of HEU with the highest scores, compared to 9.4% of HUU (Fig 1). The risk score had a nonparametric distribution and many subjects scored 0.

For peer review only - http://bmjopen.bmj.com/site/about/guidelines.xhtml

Discussion

In our prospective cohort of children presenting with WHO IMCI criteria for clinical pneumonia in the post-PCV era, the most common clinical features were crackles, nasal flaring, and lower chest wall indrawing. Despite the clinical diagnosis of pneumonia, features were heterogeneous. Thirty-four percent had an oxygen requirement and 2.1% needed CPAP support, though with best available treatment, overall mortality was low – and possibly a reflection of improvements in child health overall as a result of antiretroviral and antimalarial therapies, vaccination, and community management of nutrition. Ten percent of children hospitalised with pneumonia were HEU and this group was significantly younger, with lower birth weight and anthropometric parameters. HEU children had a significantly higher prevalence of vomiting, age-defined tachypnoea in infancy, and higher RISC scores, compared to HUU children.

Though HEU children were significantly younger and had significantly lower MUAC than HUU children, and therefore age and anthropometry are potential triage indicators for use in the clinical management of pneumonia, these differences were subtle and may not be useful for a clinician, who in low resource settings make decisions primarily on clinical suspicion. WHO IMCI clinical criteria for pneumonia are non-specific, and based on presence of cough, fast and difficult breathing. The non-specific nature of the diagnosis, which covers both infectious and non-infectious causes, is deliberately intended to increase sensitivity; however, as reflected in this study, this may have resulted in the term being used with a variety of features. Much research has been expended on developing pneumonia risk scores to identify children at risk of severe disease. However, the limitations of existing pneumonia risk scores for children in low-

For peer review only

and middle-income country settings [15], including Pneumonia Etiology Research in Child Health (PERCH) [16], Respiratory Syncytial Virus Network (ReSViNet) [17], RISC [14], mRISC [12], and RISC-Malawi [13], is that the predictive capacity is generally modest and, in the case of PERCH, was found to be not as discriminatory as the WHO danger signs. The risk scores we calculated in our cohort were not normally distributed and yielded surprisingly divergent results, indicating that they are limited to their study populations and are not generalisable.

In our cohort, the lack of predominating features of clinical pneumonia points to another possibility that, in post-PCV settings where HIV and chronic malnutrition rates are high (10% and 39%, respectively), children do not present with typical signs and symptoms. The difference in distribution of risk scores within our cohort highlights the fact that risk scores are population- and cohort-specific. In our cohort a substantial proportion of children met criteria for severe acute malnutrition (9.7% had weight-for-height z scores ≤3; and 4.1% had MUAC <11.5 cm), indicating how widespread chronic malnutrition is in Malawi. This pervasive underlying comorbidity leads to subsequent issues with identification and management of disease; and may in part explain overuse of antimicrobials in this setting, one factor contributing to an increase in antimicrobial resistance.

Antimicrobial stewardship and infection control issues in low-resource settings have been particularly problematic among young children, with significant increases in antimicrobial resistance noted among young infants with bloodstream infections over a twenty-year period [5], and concerns over resistance rates in childhood pneumonia in sub-Saharan Africa [18].

For peer review only

For peer review only - http://bmjopen.bmj.com/site/about/guidelines.xhtml

Challenges with diagnostic capacity and the limitations of clinical diagnosis hinder appropriate antimicrobial stewardship [19]. Risk scores that are adequately discriminatory therefore present an opportunity for targeted interventions, including early aggressive support, as well as judicious allocation of resources.

This study has several limitations. Only a very small proportion of HIV-exposed infants had HIV status tested by PCR, as recommended by national guidelines. This was due to shortage of HIV counselors, lack of testing reagents and reluctance of parents to obtain additional testing on their children. We did not recruit moribund children who died very soon after arrival, and therefore we may have underestimated our fatality rate. Demographics may play into admissions at a government referral tertiary hospital, and therefore there may be selection bias for sicker children to present. A substantial proportion of recruited participants had incomplete files and were therefore not included in the analysis. Due to small numbers, we were unable to calculate predictive values for severe outcomes. However, this was a detailed prospective study conducted in a low resource setting in the post-PCV era where robust diagnostic testing was available.

In conclusion, in this setting where prevalence of HIV and malnutrition is high, children hospitalised fulfilling the WHO IMCI criteria for clinical pneumonia in the post-PCV era present with heterogeneous features. These vary by HIV exposure status but this does not influence either the frequency of danger signs or mortality. The sample size limited our ability to calculate predictive scores; and the performance of available severity scores in this population and the absence of more specific diagnostics hinder appropriate antimicrobial stewardship and the rational application of other interventions. Further work in developing antimicrobial stewardship

For peer review only

For peer review only - http://bmjopen.bmj.com/site/about/guidelines.xhtml

scores in resource-limited settings, in a period defined by rising rates of antimicrobial resistance, may be warranted.

1,990 words

Acknowledgements

The authors wish to thank the collaborating members of the VacSurv Consortium (James Beard, Amelia C Crampin, Carina King, Sonia Lewycka, Hazzie Mvula, Tambosi Phiri, Miren Iturriza-Gomara, Osamu Nakagomi, Jennifer Verani, Cynthia Whitney).

Funding statement

This work was supported by a Wellcome Trust Programme Grant (Grant number 091909/Z/10/Z) and the MLW Programme Core Award (Grant 206454) from the Wellcome Trust. The funders had no role in study design, data collection and analysis, decision to publish, or preparation of the manuscript. The findings and conclusions in this report are those of the authors and do not necessarily represent the official position of the Centers for Disease Control and Prevention, Atlanta USA.

Competing interests

PI has received investigator-initiated research grant support from Bill & Melinda Gates Foundation outside the scope of this work; NAC has received research grant support and honoraria for participation in rotavirus vaccine advisory board meetings from GlaxoSmithKline

For peer review only

Biologicals and from Takeda Pharmaceuticals outside the scope of this work; NF has received investigator-initiated research grant support from GlaxoSmithKline Biologicals and from Takeda Pharmaceuticals outside the scope of this work. NBZ has received investigator-initiated research grant support from GlaxoSmithKline Biologicals, Takeda Pharmaceuticals, Merck-Sharpe-Dohme and the Serum Institute of India, all outside the scope of this work. All other authors declare that they have no financial disclosures or competing interests.

Author contributorship statement

Conceived and designed the research question: PI, NBZ; Conducted the statistical analysis: JC, MH; Managed the study: LN, IM; Developed and awarded funding for the study: DE, NAC, CM, NF, RH; Wrote the first draft of the manuscript: PI; Provided critical feedback on the manuscript: JC, MH, NAC, NF, RH, NBZ; Reviewed and approved the final draft: all authors.

Data availability

Data is available upon reasonable request to the corresponding author.

Ethics statement

This study was approved by the University of Malawi College of Medicine Research Ethics Committee (P.05/13/1386) and by the University of Liverpool (RETH490).

For peer review only

References

1. UNAIDS: **Malawi factsheet**. 2019.

2. World Bank: **Prevalence of stunting - Malawi**. 2018.

3. WHO: **Malawi Maternal and Child Health Data**. Geneva, Switzerland: WHO; 2015.

4. WHO: **Revised WHO classification and treatment of childhood pneumonia at health facilities**. Geneva, Switzerland: WHO; 2014.

5. Iroh Tam PY, Musicha P, Kawaza K, Cornick J, Denis B, Freyne B, Everett D, Dube Q, French N, Feasey N *et al*: **Emerging Resistance to Empiric Antimicrobial Regimens for Pediatric Bloodstream Infections in Malawi (1998-2017)**. *Clin Infect Dis* 2019, **69**(1):61-68.

6. Landes M, van Lettow M, Chan AK, Mayuni I, Schouten EJ, Bedell RA: **Mortality and health outcomes of HIV-exposed and unexposed children in a PMTCT cohort in Malawi**. *PLoS One* 2012, **7**(10):e47337.

7. Slogrove A, Reikie B, Naidoo S, De Beer C, Ho K, Cotton M, Bettinger J, Speert D, Esser M, Kollmann T: **HIV-exposed uninfected infants are at increased risk for severe infections in the first year of life**. *J Trop Pediatr* 2012, **58**(6):505-508.

For peer review only - http://bmjopen.bmj.com/site/about/guidelines.xhtml

8. Slogrove AL, Goetghebuer T, Cotton MF, Singer J, Bettinger JA: **Pattern of Infectious Morbidity in HIV-Exposed Uninfected Infants and Children**. *Front Immunol* 2016, **7**:164.

9. Zar HJ, Barnett W, Stadler A, Gardner-Lubbe S, Myer L, Nicol MP: **Aetiology of childhood pneumonia in a well vaccinated South African birth cohort: a nested case-control study of the Drakenstein Child Health Study**. *Lancet Respir Med* 2016, **4**(6):463-472.

10. Malawi Ministry of Health: **Clinical Management of HIV in Adults and Children**. Malawi; 2011.

11. WHO: **Handbook IMCI. Integrated management of childhood illness**. In. Geneva, Switzerland: World Health Organization; 2000.

12. Emukule GO, McMorrow M, Ulloa C, Khagayi S, Njuguna HN, Burton D, Montgomery JM, Muthoka P, Katz MA, Breiman RF *et al*: **Predicting mortality among hospitalized children with respiratory illness in Western Kenya, 2009-2012**. *PLoS One* 2014, **9**(3):e92968.

13. Hooli S, Colbourn T, Lufesi N, Costello A, Nambiar B, Thammasitboon S, Makwenda C, Mwansambo C, McCollum ED, King C: **Predicting Hospitalised Paediatric Pneumonia Mortality Risk: An External Validation of RISC and mRISC, and Local Tool Development (RISC-Malawi) from Malawi**. *PLoS One* 2016, **11**(12):e0168126.

14. Reed C, Madhi SA, Klugman KP, Kuwanda L, Ortiz JR, Finelli L, Fry AM: **Development of the Respiratory Index of Severity in Children (RISC) score among young children with respiratory infections in South Africa**. *PLoS One* 2012, **7**(1):e27793.

For peer review only

For peer review only - http://bmjopen.bmj.com/site/about/guidelines.xhtml

15. Deardorff KV, McCollum ED, Ginsburg AS: **Pneumonia Risk Stratification Scores for Children in Low-Resource Settings: A Systematic Literature Review**. *Pediatr Infect Dis J* 2018, **37**(8):743-748.

16. Gallagher KE, Knoll MD, Prosperi C, Baggett HC, Brooks WA, Feikin DR, Hammitt LL, Howie SRC, Kotloff KL, Levine OS *et al*: **The Predictive Performance of a Pneumonia Severity Score in Human Immunodeficiency Virus-negative Children Presenting to Hospital in 7 Low- and Middle-income Countries**. *Clin Infect Dis* 2020, **70**(6):1050-1057.

17. Justicia-Grande AJ, Pardo-Seco J, Cebey-Lopez M, Vilanova-Trillo L, Gomez-Carballa A, Rivero-Calle I, Puente-Puig M, Curros-Novo C, Gomez-Rial J, Salas A *et al*: **Development and Validation of a New Clinical Scale for Infants with Acute Respiratory Infection: The ReSVinet Scale**. *PLoS One* 2016, **11**(6):e0157665.

18. Iroh Tam PY, Sadoh AE, Obaro SK: **A meta-analysis of antimicrobial susceptibility profiles for pneumococcal pneumonia in sub-Saharan Africa**. *Paediatr Int Child Health* 2018, **38**(1):7-15.

19. Iroh Tam PY: **The Challenge and Opportunity of Pediatric Antimicrobial Stewardship in Low Resource Settings**. *J Trop Pediatr* 2020, **66**(1):1-3.

For peer review only

Table 1. List of variables used to compute RISC scores

**RISC**	**Score**
Oxygen saturation ≤90%	3
Chest indrawing	2
Refusal to feed	1
Wheezing	-2
WHO weight for age z-score ≤ -3	2
WHO weight for age z-score -3 ≤_z < -2	1

RISC, Respiratory Index of Severity in Children

For peer review only

For peer review only - http://bmjopen.bmj.com/site/about/guidelines.xhtml

Table 2. Characteristics of children hospitalised with clinical pneumonia, stratified by HIV exposure status

		Group		
Characteristics	All (N=1139)	HEU (N=101)	HUU (N=1038)	p-value
**Demographic and physiological characteristics**				
Age, months (m)				0.02†
N	1118	99	1019	
Median (IQR)	11.0 (6.0, 20.0)	9.0 (5.0, 15.5)	11.0 (6.0, 20.0)	
Sex				0.92*
N	1139	101	1038	
Male (%)	677 (59.4)	61 (60.4)	616 (59.3)	
Birth weight, kg				0.03†
N	1117	97	1020	
Median (IQR)	3.0 (2.3, 3.3)	2.8 (1.8, 3.2)	3.0 (2.4, 3.3)	
Weight for height				< 0.01¥
N	1098	100	998	
Mean (SD)	-0.7 (2.5)	-1.2 (2.5)	-0.6 (2.5)	
MUAC, cm				< 0.01†
N	1129	101	1028	
Median (IQR)	14.0 (13.0, 15.0)	13.6 (12.6, 14.6)	14.0 (13.0, 15.0)	
Temperature				0.02†
N	1133	100	1033	
Median (IQR)	37.8 (36.9, 38.6)	38.0 (37.2, 39.0)	37.8 (36.8, 38.5)	
Fever				0.06
N	1133	100	1033	
Yes (%)	518 (45.7)	55 (55.0)	463 (44.8)	
Antibiotic use				0.34*
N	1131	101	1030	
Yes (%)	451 (39.9)	45 (44.6)	406 (39.4)	
Inability to drink				0.08*
N	1134	101	1033	
Yes (%)	91 (8.0)	13 (12.9)	78 (7.6)	
Vomiting				0.02*
N	1137	101	1036	

For peer review only

Yes (%)	261 (23.0)	33 (32.7)	228 (22.0)	
At least one IMCI danger sign				0.05*
N	1139	101	1038	
Yes (%)	424 (37.2)	47 (46.5)	377 (36.3)	
**Clinical features**				
Nasal flaring				0.92*
N	1137	101	1036	
Yes (%)	608 (53.5)	55 (54.5)	553 (53.4)	
Lower chest wall indrawing				1.00*
N	1137	101	1036	
Yes (%)	605 (53.2)	54 (53.5)	551 (53.2)	
Stridor				0.44*
N	1137	101	1036	
Yes (%)	22 (1.9)	3 (3.0)	19 (1.8)	
Age-defined tachypnoea				0.02*
N	666	57	609	
Age 0-12m and >50 breaths/min	342 (51.4)	39 (68.4)	303 (49.8)	
Age 12-60m and >40 breaths/min	324 (48.7)	18 (31.6)	306 (50.3)	
Oral thrush/sores				0.11*
N	1138	101	1037	
Yes (%)	21 (1.9)	4 (4.0)	17 (1.6)	
Hypoxemia <90%				0.40*
N	1128	100	1028	
Yes (%)	286 (25.4)	29 (29.0)	257 (25.0)	
**Risk scores**				
RISC				<0.01*
N	1120	100	1020	
-2 to 2	1004 (89.6)	80 (80.0)	924 (90.6)	
3-6	116 (10.4)	20 (20.0)	96 (9.4)	
**Clinical management**				
Received oxygen				0.04*
N	1129	100	1029	
Yes (%)	383 (33.9)	44 (44.0)	339 (32.9)	
Placed on CPAP				0.14*
N	1118	99	1019	
Yes (%)	23 (2.1)	4 (4.0)	19 (1.9)	
**Outcomes**				
Length of hospital stay, days				0.21†
N	1086	96	990	
Median (IQR)	2.0 (2.0, 4.0)	2.0 (2.0, 4.0)	2.0 (2.0, 3.0)	

For peer review only - http://bmjopen.bmj.com/site/about/guidelines.xhtml

Mortality				0.07*
N	1139	101	1038	
Yes (%)	5 (0.4)	2 (2.0)	3 (0.3)	

CPAP, continuous positive airway pressure; HEU, HIV-exposed, uninfected; HUU, HIV-unexposed, uninfected; IMCI, Integrated Management of Childhood Illness; IQR, interquartile range; MUAC, mid-upper arm circumference; RISC, Respiratory Index of Severity in Children

** Fisher's exact test*

† Wilcoxon rank sum test

¥ Independent two sample t-test

For peer review only

Figure legend

Fig 1. Summary of RISC scores computed from the data

RISC, Respiratory Index of Severity in Children

Appendix

Supplementary Table 1. STROBE checklist

Supplementary Table 2. Full characteristics of children hospitalised with clinical pneumonia, stratified by HIV exposure status

For peer review only

For peer review only - http://bmjopen.bmj.com/site/about/guidelines.xhtml

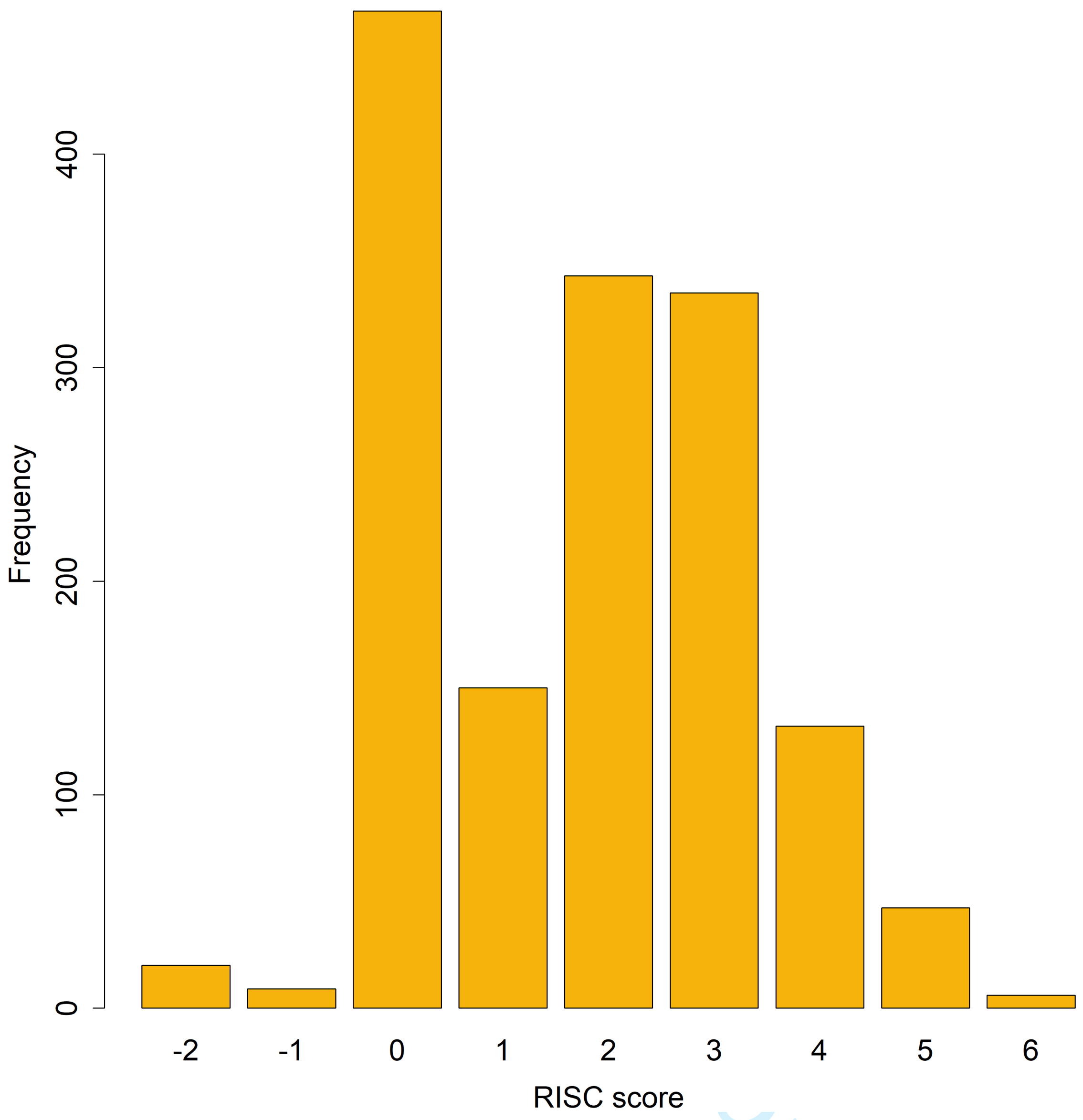
Frequency
0
100
200
300
400
-2
-1
0
1
2
3
4
5
6
RISC score

Supplementary Table 1. STROBE Statement—Checklist of items that should be included in reports of ***cohort studies***

	Item No	Recommendation	Page
**Title and abstract**	1	(*a*) Indicate the study's design with a commonly used term in the title or the abstract	1
		(*b*) Provide in the abstract an informative and balanced summary of what was done and what was found	3
**Introduction**			
Background/rationale	2	Explain the scientific background and rationale for the investigation being reported	6
Objectives	3	State specific objectives, including any prespecified hypotheses	7
**Methods**			
Study design	4	Present key elements of study design early in the paper	7
Setting	5	Describe the setting, locations, and relevant dates, including periods of recruitment, exposure, follow-up, and data collection	7
Participants	6	(*a*) Give the eligibility criteria, and the sources and methods of selection of participants. Describe methods of follow-up	7
		(*b*) For matched studies, give matching criteria and number of exposed and unexposed	N/A
Variables	7	Clearly define all outcomes, exposures, predictors, potential confounders, and effect modifiers. Give diagnostic criteria, if applicable	8
Data sources/ measurement	8*	For each variable of interest, give sources of data and details of methods of assessment (measurement). Describe comparability of assessment methods if there is more than one group	8
Bias	9	Describe any efforts to address potential sources of bias	9
Study size	10	Explain how the study size was arrived at	9
Quantitative variables	11	Explain how quantitative variables were handled in the analyses. If applicable, describe which groupings were chosen and why	9
Statistical methods	12	(*a*) Describe all statistical methods, including those used to control for confounding	9
		(*b*) Describe any methods used to examine subgroups and interactions	9
		(*c*) Explain how missing data were addressed	9
		(*d*) If applicable, explain how loss to follow-up was addressed	9
		(*e*) Describe any sensitivity analyses	N/A
**Results**			
Participants	13*	(a) Report numbers of individuals at each stage of study—eg numbers potentially eligible, examined for eligibility, confirmed eligible, included in the study, completing follow-up, and analysed	9
		(b) Give reasons for non-participation at each stage	9
		(c) Consider use of a flow diagram	N/A

For peer review only - http://bmjopen.bmj.com/site/about/guidelines.xhtml

Descriptive data	14*	(a) Give characteristics of study participants (eg demographic, clinical, social) and information on exposures and potential confounders	Table 2
		(b) Indicate number of participants with missing data for each variable of interest	9
		(c) Summarise follow-up time (eg, average and total amount)	9
Outcome data	15*	Report numbers of outcome events or summary measures over time	9
Main results	16	(*a*) Give unadjusted estimates and, if applicable, confounder-adjusted estimates and their precision (eg, 95% confidence interval). Make clear which confounders were adjusted for and why they were included	9-10
		(*b*) Report category boundaries when continuous variables were categorized	Table 2
		(*c*) If relevant, consider translating estimates of relative risk into absolute risk for a meaningful time period	N/A
Other analyses	17	Report other analyses done—eg analyses of subgroups and interactions, and sensitivity analyses	N/A
**Discussion**			
Key results	18	Summarise key results with reference to study objectives	10-11
Limitations	19	Discuss limitations of the study, taking into account sources of potential bias or imprecision. Discuss both direction and magnitude of any potential bias	13
Interpretation	20	Give a cautious overall interpretation of results considering objectives, limitations, multiplicity of analyses, results from similar studies, and other relevant evidence	13
Generalisability	21	Discuss the generalisability (external validity) of the study results	13
**Other information**			
Funding	22	Give the source of funding and the role of the funders for the present study and, if applicable, for the original study on which the present article is based	14

*Give information separately for exposed and unexposed groups.

Note: An Explanation and Elaboration article discusses each checklist item and gives methodological background and published examples of transparent reporting. The STROBE checklist is best used in conjunction with this article (freely available on the Web sites of PLoS Medicine at http://www.plosmedicine.org/, Annals of Internal Medicine at http://www.annals.org/, and Epidemiology at http://www.epidem.com/). Information on the STROBE Initiative is available at http://www.strobe-statement.org.

For peer review only - http://bmjopen.bmj.com/site/about/guidelines.xhtml

Supplementary Table 2. Full characteristics of children hospitalised with clinical pneumonia, stratified by HIV exposure status

		Group		
Characteristics	All (N=1139)	HEU (N=101)	HUU (N=1038)	p-value
**Demographic and physiological characteristics**				
Age group				
N	1116	99	1017	
0-2 m	126 (11.3)	13 (13.1)	113 (11.1)	
2-6 m	188 (16.9)	23 (23.2)	165 (16.2)	
6-12 m	302 (27.1)	32 (32.3)	270 (26.6)	
12-24 m	318 (28.5)	18 (18.2)	300 (29.5)	
24-60 m	182 (16.3)	13 (13.1)	169 (16.6)	
Low birth weight				0.04*
N	1117	97	1020	
< 2.5 kg (%)	302 (27.0)	35 (36.1)	267 (26.2)	
Weight for height categories				
N	1098	101	998	
<-3 (%)	106 (9.7)	15 (15. 0)	91 (9.1)	
-3- -2 (%)	248 (0.9)	31 (31.0)	217 (21.7)	
>-2 (%)	1102 (98.6)	54 (54.0)	690 (69.1)	
MUAC categories				
N	1139	101	1038	
< 11.5 cm (%)	47 (4.1)	13 (12.9)	34 (3.3)	
Previous visit to health centre				1.00*
N	1139	101	1038	
Yes (%)	979 (86.0)	87 (86.1)	892 (85.9)	
**Household characteristics**				
Household size >4				0.83*
N	1136	101	1035	
Yes (%)	480 (42.3)	44 (43.6)	436 (42.1)	
**Clinical features**				
Crackles				0.75*
N	1139	101	1038	
Yes (%)	623 (54.7)	57 (56.4)	566 (54.5)	
Wheezing				0.41*
N	1137	101	1036	
Yes (%)	192 (16.9)	20 (19.8)	172 (16.6)	
Bronchial breathing				0.12*
N	1139	101	1038	
Yes (%)	70 (6.2)	10 (9.9)	60 (5.8)	
Reduced air entry				0.19*
N	1139	101	1038	

For peer review only

Yes	9 (0.8)	2 (2.0)	7 (0.7)	
Age-adjusted tachycardia				0.11*
N	505	46	459	
Age 0-12m and >160 beats/min	266 (52.7)	31 (67.4)	235 (51.2)	
Age 12-36m and >150 beats/min	204 (40.4)	14 (30.4)	190 (41.4)	
Age 36-60m and >140 beats/min	35 (6.9)	1 (2.2)	34 (7.4)	
Hepatomegaly				0.61*
N	1135	99	1036	
Yes (%)	11 (1.0)	0 (0.0)	11 (1.1)	
Oxygen saturation				0.28†
N	1128	100	1028	
Median (IQR)	95.0 (89.0, 98.0)	95.0 (86.0, 98.0)	95.0 (89.8, 98.0)	
**Clinical management**				
Received IVF				1.00*
N	1120	100	1020	
Yes (%)	14 (1.3)	1 (1.0)	13 (1.3)	

HEU, HIV-exposed, uninfected; HUU, HIV-unexposed, uninfected; IQR, interquartile range; IVF, intravenous fluids; MUAC, mid-upper arm circumference

* *Fisher's exact test*

† Wilcoxon rank sum test

For peer review only
